# Retrofitting language models to operate over bytes

**DOI:** 10.1038/s41586-026-11111-4

**Published:** 2026-10-07

**Authors:** Benjamin Minixhofer, Tyler Murray, Tomasz Limisiewicz, Anna Korhonen, Luke Zettlemoyer, Noah A. Smith, Edoardo M. Ponti, Luca Soldaini, Valentin Hofmann

**Affiliations:** 1 Allen Institute for AI, Seattle, WA USA; 2 Language Technology Lab, University of Cambridge, Cambridge, UK; 3 University of Washington, Seattle, WA USA; 4 Imperial College London, London, UK; 5 LMU Munich, Munich, Germany; 6 Munich Center for Machine Learning, Munich, Germany

**Keywords:** Computer science, Information technology

## Abstract

Recent advances in artificial intelligence (AI) have largely been driven by large language models, deep neural networks that operate over discrete units called tokens. To represent text, most large language models use words or word fragments as the tokens, known as subword tokenization^1^. Subword tokenization obscures fine-grained information, which is problematic, especially for scientific data—such as computer code or biological sequences—where meaning depends on the individual characters or bytes^2^. Models that instead operate directly on the byte encoding of text avoid these limitations, but until now they have lagged behind subword-based models in performance. Here we introduce a general method for creating byte-level large language models through byteification that approach the capabilities of subword-based systems. We use a two-stage conversion procedure to retrofit existing subword-based models into byte-level models with minimal extra training. The resulting models outperform earlier byte-level approaches and excel on character-level reasoning tasks, achieving practical inference speeds by efficiently processing byte-level information and adaptability by reusing the existing ecosystem around the source large language model. Our results remove a long-standing performance barrier to end-to-end byte-level language modelling, demonstrating that models operating on raw text encodings can scale competitively while offering advantages in domains requiring fine-grained textual understanding.

## Abstract (web summary)

Byteification is a general method for retrofitting large language models to operate over bytes such that they approach the capabilities of subword-based systems and may be better for scientific data.

## Main

Recent progress in AI has been driven by end-to-end deep learning systems that learn representations directly from data. Large language models (LLMs) exemplify this trend, achieving strong capabilities by training on massive collections of text^3,4^. However, despite their apparent generality, contemporary LLMs are not fully end-to-end: before learning can begin, text must first be mapped to a sequence of discrete units called tokens. The choice of tokens, although sometimes overlooked, fundamentally shapes the representations that LLMs learn and the behaviours that they exhibit^5–9^.

The vast majority of contemporary LLMs use words or parts of words as the tokens in a process known as subword tokenization^1,10^. This leads to many problems. LLMs that use subword tokenization suffer from limited character-level understanding^11–13^, which especially hinders performance with scientific data, such as code and biological sequences^2,14–17^; they are also implicitly biased towards generating particular responses based on how the prompt is tokenized^18–20^, they are restricted in the number of words they can incorporate in their vocabulary, which in practice leads to English-centricity^6,21^, and they potentially suboptimally allocate their compute^2,22^. These problems have motivated extensive research into alternatives to subword tokenization, most commonly by using the underlying UTF-8 bytes that the text is encoded as^23^ as the discrete units. Many earlier byte-level LLMs claim to outperform subword-level LLMs on the efficiency–performance Pareto frontier^2,22,24–27^. However, in practice, byte-level LLMs have not seen widespread adoption so far, and all leading LLMs still exclusively rely on subword tokenization.

We hypothesize that the key reason for this mismatch between theory and practice is that existing approaches to byte-level language modelling focus predominantly on training a new byte-level model from a random initialization and comparing it against a subword-level LLM also trained from a random initialization. By contrast, the training of state-of-the-art subword-level LLMs is rapidly evolving, combining innovations in training data curation, model architecture and post-training. Keeping up with this pace is infeasible for byte-level LLM development without extensive investments.

To resolve this mismatch, we introduce a general method for creating byte-level LLMs that achieve performance close to that of state-of-the-art subword-level LLMs across various tasks, while vastly surpassing earlier byte-level LLMs with a comparable parameter count. In contrast to earlier byte-level LLMs that focus predominantly on training from random initialization, we retrofitted an existing subword-level LLM to the byte level using less than 1% of a typical pretraining budget (49.1B tokens in total, where B represents a billion or 10^9^). We refer to this process as byteification. Byteification establishes a connection between existing subword-level LLMs and byte-level LLMs. This let us train the byteified models Bolmo 7B and Bolmo 1B by starting from the existing fully open LLMs Olmo 3 7B^28^ and OLMo 2 1B^29^, Bwen 8B by byteifying Qwen3 8B Base^30^, and Blama 8B by byteifying Llama 3 8B^31^.

Our architecture first aggregates the byte stream into a sequence of patches, where each patch itself consists of one or more bytes. The patches are then processed by a large transformer^32^ language model and finally depooled into bytes. Owing to its latent tokenization into byte patches, we refer to this style of architecture as a latent tokenizer language model (LTLM). Examples of LTLMs include the DTP^24^, BLT^22^ and H-Net^2^ models. However, in contrast to earlier work, we specifically designed our architecture to be well suited to byteification (section ‘Byteified language model architecture’). In particular, we resolved a mismatch between the expressivity of subword tokenization and the latent LTLM tokenization. Alongside an efficient two-stage training procedure (section ‘Byteification training procedure’), this allowed us to quickly recover and, in some cases, surpass the performance of the source subword-level LLM. We believe that byteifying provides a key missing research direction by enabling the creation of state-of-the-art byte-level LLMs without extensive investment. This approach is complementary to training from random initialization. Making it cheap to byteify any subword model can quickly unveil high-performing architectures that are promising candidates for training from random initialization as byte-level LLMs.

Our byteified models outperformed, on average, all earlier publicly available byte-level LLMs of comparable size. For example, Bolmo 7B achieved a +16.5% absolute improvement in STEM tasks over BLT 7B, which was trained from random initialization. Bolmo 7B also greatly outperformed the source Olmo 3 on character understanding and was better in certain coding settings. Bwen 8B outperformed Bolmo 7B, exhibiting comparable trends and reaching performance close to and sometimes surpassing the source Qwen model. Furthermore, we found that the byteified models can arbitrarily be further sped up by training with higher ratios of bytes per patch, which is possible only to a limited extent in subword-level LLMs. Finally, we show that existing components in the source LLM ecosystem can be used to adapt a byteified model without any extra training cost; this could further accelerate research on byte-level LLMs.

In aggregate, our results show that byte-level LLMs offer substantial promise as a foundation for future language models, with potential advantages for computational efficiency by reducing energy and deployment costs, for reducing biases introduced by English-centric subword tokenization and for enabling applications that require fine-grained textual understanding, particularly in scientific and technical domains.

### Byteified language model architecture

**Fig. 1:**
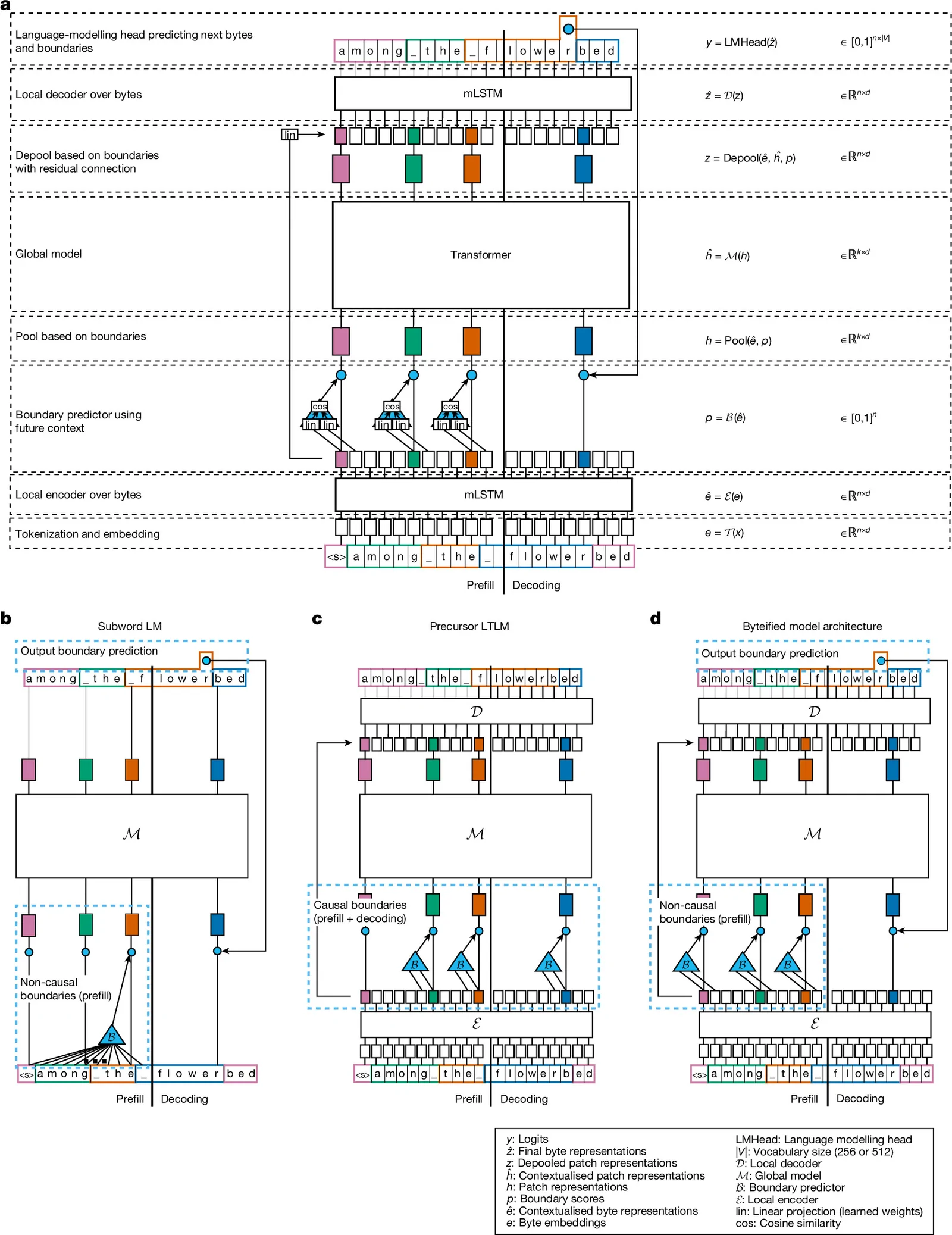
Our byteified model architecture and boundary prediction procedure in contrast with earlier work. **a**, Bolmo architecture: ${\mathcal{T}}$, the tokenization and embedding operation, transforms the input text into one representation per byte. The representations are contextualized with the local encoder ${\mathcal{E}}$ consisting of mLSTM blocks^33^. The boundary predictor ${\mathcal{B}}$ decides where to place patch boundaries using 1 byte of future context. The representations are then pooled, passed through the global transformer model ${\mathcal{M}}$ and depooled. Finally, the local decoder ${\mathcal{D}}$, which consists of another mLSTM stack, contextualizes the depooled byte representations and the language-modelling head transforms them into next-byte predictions and decides where to place the next patch boundary. **b**, Subword-level LLMs non-causally set boundaries over the prefill using the external subword tokenizer and then implicitly predict boundaries and the text content during decoding. **c**, Earlier byte-level LTLMs causally set boundaries with a lightweight boundary predictor during both prefill and decoding. **d**, Our byteification architecture restores the expressivity of subword-level LLM boundaries by non-causally predicting boundaries for the prefill and then predicting during decoding whether a boundary occurs and the next byte.

**Extended Data Fig. 1:**
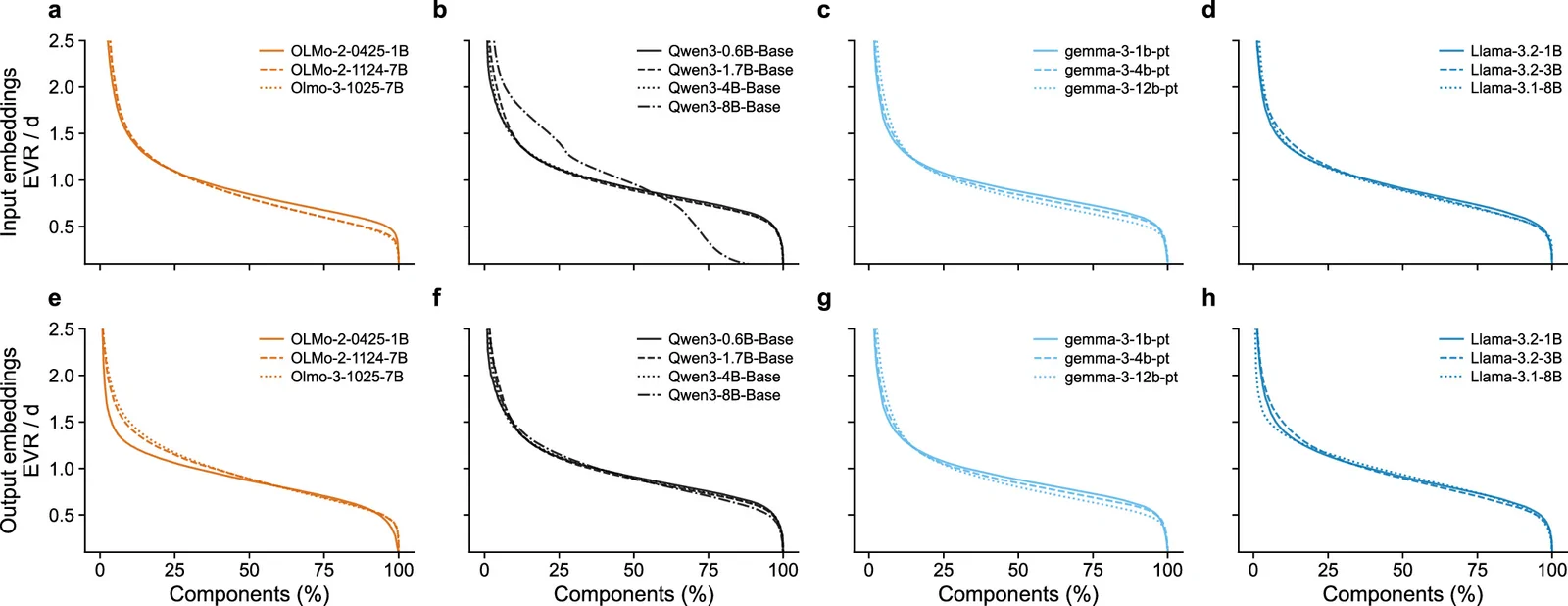
Explained variance ratio of the singular values of the input and output embedding matrices. **a-d**, Input embedding explained variance ratios acros model families. **e-h**, Output embedding explained variance ratios across model families. Variance ratios are normalized by the number of dimensions; they decay smoothly with the number of components, up until a steep dropoff toward the highest-rank components. This indicates that it is difficult to approximate the embedding matrices using lower-rank structure. A notable exception is Qwen3-4B-Base, which may be more amenable to a lower-dimensional local encoder.

Following the same overall structure as earlier LTLMs (‘Related work’ in Methods), the byteified models can be formalized as shown in Fig. 1. In a nutshell, a local encoder model operating over bytes first creates contextualized byte representations. A neural boundary predictor module then predicts, based on these representations, the byte positions at which to place a boundary. The boundary information is used to derive a sequence of patches: a patch is the contiguous sequence of bytes between consecutive boundaries (including the byte where the end boundary is placed). A pooling module then reduces the contextualized byte representations to a single representation per patch, which is passed through a deep global model. The patch representations contextualized through the global model are depooled back to the byte level and further contextualized through a local decoder model. Finally, the local decoder representations are used to predict the next byte and any other potential information. We chose the local models to be shallow but wide as we found that the embeddings of the original model typically have a high effective rank (Extended Data Fig. 1), and we chose matrix long short-term memory (mLSTM) layers^33^ to contextualize local information as they retained high performance at high throughput in our experiments (Extended Data Fig. 3). See ‘Byteified model architecture details’ in Methods for a detailed description of the architecture. Our primary departure from earlier architectures lies in the non-causal patch boundary prediction. The boundary predictor of earlier LTLMs uses only the past context to decide on whether to place a boundary (it is causal, referred to as incremental by Pagnoni et al.^22^). At a glance, this seems necessary: we are aiming to predict the next byte, so we must not leak any information about it. However, although subword-level LLMs use a causality constraint over the subword tokens, the subword tokens themselves do not depend exclusively on past context: subword tokenizers use information about future bytes to place token boundaries. To see this, let us interpret our subword tokenizer as a function that decides whether to place a token boundary after any byte: ${\mathcal{B}}(x):{\{0,1,\ldots ,255\}}^{n}\to {\{0,1\}}^{n}$, where 1 at the *n*th place denotes a token boundary predicted after the *n*th byte. Let us assume (1) a vocabulary of English words and subwords, (2) the example text ‘_Hello_Wor!’, which would typically be tokenized as {‘_Hello’, ‘_Wor’, ‘!’}, and (3) the position *i* = ∣‘_Hello_Wor’∣ − 1 = 9. ${\mathcal{B}}$ (‘_Hello_Wor!’)_*i*_ = 1, as there is a boundary after ‘r’. However, in the text ‘_Hello_World!’, which would be tokenized as {‘_Hello’, ‘_World’, ‘!’}, we have ${\mathcal{B}}$ (‘_Hello_World!’)_*i*_ = 0, despite ‘_Hello_Wor!’[:*i *+ 1] = ‘_Hello_World!’[:*i *+ 1] = ‘_Hello_Wor’. In other words, although the subword-level LLM uses only past subword tokens to predict the next subword token, the subword tokens themselves are created by taking future context into account. In this case, this means deciding that ‘_Wor’ should be a token in one case but not in the other, although the text up until that point is equivalent. Current LTLMs, in contrast, cannot take future context into account. This creates a mismatch between the expressivity of LTLM boundary predictors and subword tokenizers. We modified the boundary predictor to resolve this mismatch. In particular, although earlier boundary predictors can be written as

$${\mathcal{B}}{(\hat{e})}_{t}:= \,f({\hat{e}}_{0},{\hat{e}}_{1},\ldots ,{\hat{e}}_{t}),$$

with *f* a function mapping the byte representations $\hat{e}$ to boundary scores by using context up to the current byte representation ${\hat{e}}_{t}$ (where *t* is the index of the current byte), we set our boundary predictor to

$${{\mathcal{B}}}_{\mathrm{byteify}}{(\hat{e})}_{t}:= \,f({\hat{e}}_{0},{\hat{e}}_{1},\ldots ,{\hat{e}}_{t},{\hat{e}}_{t+1}).$$

That is, we use up to 1 byte of future context. Concretely, we parameterize our boundary predictor as

$${{\mathcal{B}}}_{\mathrm{byteify}}{(\hat{e})}_{t}:= \,\frac{1}{2}\left(1-\frac{{({W}_{q}{\hat{e}}_{t+1})}^{{\rm{T}}}({W}_{k}{\hat{e}}_{t})}{\parallel {W}_{q}{\hat{e}}_{t+1}\parallel \parallel {W}_{k}{\hat{e}}_{t}\parallel }\right)\in [0,1],$$

where *W*^*q*^ and *W*^*k*^ are learned projection matrices as in Hwang et al.^2^, although we compute the cosine distance between a projection of the representation of the current byte and the byte one position in the future. Even though subword tokenizers in principle have unrestricted access to the future, in practice, we found 1 byte of lookahead largely sufficient to match the behaviour of subword tokenization. As shown in Fig. 1, taking future context into account can also make patches more semantically coherent. For example, in texts containing compounds, such as ‘the flowerbed’, a boundary predictor has three intuitive options: (1) make the entire compound a single patch ‘flowerbed’, (2) place a patch boundary after ‘r’ to create the patch ‘flower’ or (3) place a patch boundary after ‘b’ (once it is evident that this is a compound word) to create ‘flowerb’. Option (2) is arguably the semantically most coherent^34^; however, for a causal boundary predictor, this would mean having to place a patch boundary after ‘r’ for every text starting with ‘the flower’, including, for example, ‘the flowers’, whereas a non-causal one can adjust based on future context. The byteification strategy of Hwang et al.^2^ applies supervision based on option (3), that is, predicting the start of the next subword token instead of the end of the previous one, which would create a patch ‘flowerb’, as shown in Fig. 1.

Although using future context is fine for prefilling, we need to know whether to place a boundary without observing the next byte for decoding. We, thus, add a special symbol <b> to the vocabulary and let the local decoder learn to emit <b> at the end of every patch (the local encoder, by contrast, never sees <b>). This is analogous to the output boundary prediction in Fleshman and Van Durme^35^. In effect, we end up with two boundary predictors: the boundary predictor ${\mathcal{B}}$ ingesting the shallowly contextualized representations from the local encoder with future context (used during prefill) and a boundary predictor as part of the language-modelling head ingesting deeply contextualized representations from the local decoder without future context (used during decoding). Notably, this is precisely equivalent to the behaviour of subword-level LLMs: the prefill is tokenized using the external subword tokenizer (analogous to the boundary predictor ${\mathcal{B}}$), and output boundaries <b> are implicitly predicted along with the text contents of every subword token upon decoding (analogous to our output boundary predictor), as illustrated in Fig. 1. This lets our byteified model achieve both accurate boundaries and representations faithful to the original model, whereas earlier approaches had to choose either one or the other (Extended Data Table 6). As we are aiming to predict the symbol <b> after every patch, our local decoder becomes a model over ${{\mathbb{R}}}^{n\times d}\to {{\mathbb{R}}}^{(n+k)\times d}$, that is, the local decoder needs to process *k* more positions. Although this overhead is not prohibitive in principle, it makes it difficult to compare models that use output boundary prediction against models that do not. We, thus, make it effectively zero-cost by doubling our byte vocabulary size from 256 to 512 for every byte by adding a version of the same byte followed by a boundary. Sampling algorithms such as top_p^36^ then act on the combined vocabulary of size 512. The goal of the local decoder, then, is to predict the current byte and whether it is followed by a boundary at every step. Fusing the boundary symbol turns the local decoder back into an isotropic model. The only remaining overhead is due to the softmax over a set of 512 instead of 256 output tokens, which is negligible.

Byteification keeps the total parameter count close to the parameter count of the source subword-level LLM, as the output embedding matrix is removed but new parameters from the local models are added (‘Byteified model architecture details’ in Methods and Extended Data Table 5).

### Byteification training procedure

Our byteifying procedure consists of two stages. In the first stage, we aim to quickly learn weights for the local encoder, local decoder, boundary predictor and language-modelling head. To do so, we introduce a new objective to optimize towards exactly recovering the behaviour of the source subword-level LLM (‘Byteifying procedure’ in Methods). The parameters of the global model stay frozen in this stage. In the second stage, we train the entire model so that it learns to use byte-level information, while also optionally increasing the target compression ratio of bytes per patch. We used a consistent experimental set-up across all experiments (‘Experimental set-up’ in Methods); see Extended Data Table 4 for a full list of training hyperparameter values.

### Performance after byteification

We byteified the Qwen3 8B^30^, Llama 3 8B^31^ and Olmo 3 7B^28^ models, and we refer to their byteified variants as Bwen 8B, Blama 8B and Bolmo 7B, respectively. Table 1 compares these models with the source subword-level models as well as with Olmo 3 with continued training on our data mix under the training settings for Bolmo. We additionally compare against existing byte-level LLMs of comparable size: EvaByte 6.5B^27^, TFree-HAT 7B^37^ and BLT 7B^22^. Bwen 8B statistically significantly outperformed all the earlier byte-level models and numerically improved in every category except GenQA (here, it slightly trailed TFree-HAT 7B). Bwen 8B, Blama 8B and Bolmo 7B were also close to matching their respective source models. Bwen 8B outperformed Bolmo 7B due to its more capable backbone. We observed similar trends for the byteified 1B model (Extended Data Table 3) in our 1B evaluation suite (Extended Data Table 2).

**Table 1:** Performance of the byteified Bolmo 7B, Bwen 8B and Blama 8B models

	Based on Qwen3		Based on Llama 3		Based on Olmo 3			Trained from random initialization		
	Bwen 8B	Qwen3 8B	Blama 8B	Llama 3 8B	Bolmo 7B	Olmo 3 7B	Olmo 3 7B (CT)	EvaByte 6.5B	TFree-HAT 7B	BLT 7B
Number of parameters (including embeddings)	8.31B	8.19B	8.25B	8.03B	7.63B	7.30B	7.30B	6.49B	7.19B	10.55B
7B byteification suite (average)	69.8	**70.3**	59.8	**60.7**	**62.5**	62.3	61.9	45.7	56.5	48.9
* P* (baseline versus Bwen 8B)	–	~0.69	–	–	–	–	–	<0.0001	<0.0001	<0.0001
* P* (baseline versus Blama 8B)	–	–	–	~0.80	–	–	–	<0.0001	~0.12	<0.0001
* P* (baseline versus Bolmo 7B)	–	–	–	–	–	~0.44	~0.29	<0.0001	<0.01	<0.0001
Char	**78.9**	66.2	**81.0**	57.2	**78.1**	54.3	70.7	43.2	49.3	46.4
CUTE	**87.2**	66.9	**87.9**	58.3	**80.9**	54.7	74.6	47.7	54.5	48.0
EXECUTE	**70.7**	65.5	**74.1**	56.2	**75.4**	53.8	66.9	38.8	44.2	44.9
Code (pass@1)	39.6	**45.8**	23.7	**29.1**	27.6	**31.1**	29.4	23.2	28.4	24.5
DS 1000 pass@1	22.0	**32.9**	14.1	**25.7**	15.3	**20.1**	19.4	7.1	18.2	17.0
HumanEval pass@1	62.9	**71.0**	32.9	**40.8**	40.6	**49.0**	42.4	34.7	41.1	31.5
DeepSeek LeetCode pass@1	5.9	**8.2**	**0.9**	0.2	**2.3**	1.6	0.7	1.6	0.9	1.2
MBPP pass@1	55.1	**66.5**	35.4	**44.4**	42.8	44.3	**45.7**	42.9	44.6	37.2
MultiPL-E HumanEval pass@1	43.5	**47.4**	24.2	**31.6**	26.8	**33.6**	30.1	16.8	26.9	24.1
MultiPL-E MBPP pass@1	48.5	**48.6**	**34.9**	31.9	38.0	37.8	**38.4**	36.5	38.8	36.0
Code (pass@16)	**69.0**	60.7	**52.0**	46.6	**56.4**	49.7	47.8	40.8	47.0	40.2
HumanEval pass@16	**89.8**	83.7	**68.5**	66.7	**74.7**	71.1	64.9	49.1	61.4	44.7
DeepSeek LeetCode pass@16	**16.4**	14.5	**4.8**	0.6	**7.6**	6.2	2.9	3.3	4.6	4.8
MBPP pass@16	**79.2**	76.1	**63.6**	62.6	**68.0**	54.9	57.2	59.2	59.2	53.2
MultiPL-E HumanEval pass@16	**82.5**	64.3	**57.6**	54.7	**62.5**	56.3	53.4	31.8	49.7	43.7
MultiPL-E MBPP pass@16	**76.9**	65.1	**65.7**	48.4	**69.2**	59.9	60.5	60.5	60.3	54.6
Math	60.2	**68.2**	30.4	**35.8**	48.9	**55.3**	49.8	27.0	35.8	15.7
GSM8K	76.7	**84.7**	47.8	**54.9**	68.0	**73.1**	67.6	36.7	60.7	24.2
MATH	43.6	**51.7**	13.0	**16.6**	29.8	**37.5**	32.0	17.3	10.9	7.3
MC_STEM_	77.0	**78.9**	64.6	**69.0**	65.5	**66.3**	66.1	54.0	62.1	49.0
ARC MC	94.4	**95.4**	83.8	**86.1**	88.5	**89.2**	88.7	74.2	90.0	65.5
MMLU STEM	74.0	**77.2**	53.6	**55.1**	57.0	**59.5**	57.7	44.3	55.2	41.6
MedMCQA MC	61.3	**63.5**	49.8	**56.9**	47.8	48.2	**48.9**	37.9	51.5	37.6
MedQA MC	59.8	**62.2**	45.7	**53.6**	42.4	42.0	**42.9**	27.2	20.5	22.5
SciQ MC	95.7	**96.0**	90.2	**93.3**	91.9	**92.8**	92.5	86.6	93.3	77.8
MC_Non-STEM_	83.0	**84.8**	73.1	**75.4**	75.8	**77.7**	76.6	63.8	66.5	56.6
MMLU Humanities	77.5	**78.6**	66.8	**70.3**	67.2	**69.2**	68.2	52.7	57.4	52.2
MMLU Social Sci.	83.3	**85.1**	71.7	**76.3**	74.0	**75.2**	**75.2**	57.4	72.0	54.0
MMLU Other	74.9	**76.6**	66.3	**69.8**	65.1	**66.9**	66.2	50.9	65.0	49.3
CSQA MC	79.2	**83.8**	69.8	**71.2**	73.6	**75.2**	74.6	91.4	75.4	52.2
PiQA MC	87.4	**89.8**	**78.0**	75.6	79.4	**80.3**	79.1	65.3	79.8	62.9
SocialIQA MC	80.9	**83.3**	74.8	**76.0**	79.1	**80.4**	79.2	79.6	79.3	50.6
CoQA Gen2MC MC	92.5	**94.1**	86.7	**88.2**	90.0	**92.9**	90.2	63.2	91.2	71.4
DROP Gen2MC MC	78.0	**78.2**	46.6	**49.4**	59.1	**62.5**	61.1	41.1	24.9	31.0
Jeopardy Gen2MC MC	90.5	**91.5**	85.1	**89.0**	84.8	85.5	**86.3**	67.8	90.5	77.5
NaturalQs Gen2MC MC	72.4	**74.1**	65.2	**69.4**	65.9	**69.6**	66.6	43.8	70.7	50.1
SQuAD Gen2MC MC	97.0	**97.2**	92.6	**93.8**	95.8	**96.8**	95.7	88.8	25.8	71.0
GenQA	70.5	**71.0**	71.0	**73.5**	70.9	**72.4**	71.7	41.4	71.3	68.4
HellaSwag RC	80.2	**80.5**	81.4	**82.0**	**78.8**	77.8	78.6	70.1	82.8	81.1
WinoGrande RC	**86.7**	86.4	86.6	**87.4**	85.5	85.7	**85.8**	78.2	88.2	88.2
Lambada	**72.4**	70.2	74.4	**75.3**	**71.1**	68.0	69.9	62.9	70.5	72.8
Basic Skills	93.2	**94.8**	87.7	**89.8**	89.6	**90.0**	89.8	82.7	89.6	84.5
DROP	**64.2**	57.2	54.1	**58.8**	65.2	**71.5**	65.0	7.8	48.6	38.8
Jeopardy	59.0	**65.1**	65.5	**72.0**	56.8	60.3	**63.1**	13.1	68.3	67.3
NaturalQs	29.9	**33.5**	32.7	**38.4**	28.6	**32.6**	31.1	5.4	34.3	29.2
SQuAD	**91.3**	89.1	88.5	**89.0**	91.6	**93.5**	92.0	35.9	88.6	85.2
CoQA	57.3	**61.6**	68.0	**69.0**	70.5	**72.7**	70.0	16.7	71.1	68.6

We compare against the source subword models (Olmo 3 7B, Qwen3 8B and Llama 3 8B), as well as Olmo 3 with continued training (CT) on our data mix and existing byte-level models of comparable size that were trained from random initialization. Bold indicates the best result within each byteified model and its source subword model(s). The byte-level models trained from random initialization are shown for reference and are excluded from this comparison. *P* values indicate the Holm–Bonferroni corrected probability (‘Statistical tests’ in Methods) of baseline performance in meeting or exceeding the performance of the byteified model over 10,000 bootstrap resamples.

For the category Code, the byteified models achieved higher pass@16 rates at lower pass@1, indicating that they generated more diverse continuations than the source subword-level models under the given sampling settings, which were equivalent for all models (temperature = 0.6 and top_p = 0.6; Extended Data Table 1). However, concluding that byte-level models are, in fact, better suited to generating more diverse generations would require mapping the quality–diversity Pareto frontier, as the observed increase in diversity could also be explained by differing effects of the sampling settings or engines on byte-level models compared with the original subword-level models. It could be useful to explore this aspect in future work (‘Future directions’ in Supplementary Information).

The byteified models substantially surpassed their subword-level counterpart regarding character understanding. By contrast, the earlier byte-level models did not outperform the subword-level models. This could be explained by character understanding being acquired primarily through scale^12^, so although byte-level models should require less scale to acquire character understanding, the increased scale of Olmo 3—probably trained on substantially more tokens than the other models—may compensate for this. Bolmo 7B, Bwen 8B and Blama 8B were trained with synthetic data that encouraged character understanding (‘Character-level training data’ in Methods), which sped up the acquisition of this skill. The Olmo 3 model with continued training on our data mix served as a control, having been trained on the same data but with a different architecture. Comparing Olmo 3 7B (CT) and Bolmo 7B, we found that in this setting, the architecture contributed to a slight degradation in the MC, GenQA and Math categories, while leading to substantial degradation in Code (pass@1) and strong improvements in Code (pass@16) as well as character-level understanding.

### Increasing model efficiency

A substantial advantage of LTLMs is that—unlike subword-level LLMs—they are not restricted to a fixed, finite set of patches. We next investigated whether we could leverage the increased freedom in our choice of patching strategy to train a faster model by encouraging a higher average number of bytes per patch. In particular, we experimented with ways to change the external boundary supervision from the original subword tokenization boundaries ${{\mathcal{B}}}_{\mathrm{subword}}$ to a subset of those boundaries selected based on byte-pair encoding (BPE)^1^, next-token entropy or cross-entropy (‘Byteifying procedure’ in Methods).

As the baseline, we increased the number of bytes per patch of the subword-level LLM through tokenizer transfer to SuperBPE tokenizers^38^. We trained SuperBPE tokenizers on top of the OLMo tokenizer to reach vocabulary sizes of {200k, 400k}, with k representing thousands, using the same 10-GB text sample as ref. ^38^ for tokenizer training. We used FOCUS^39^ to initialize the embeddings of the new superword tokens.

**Fig. 2:**
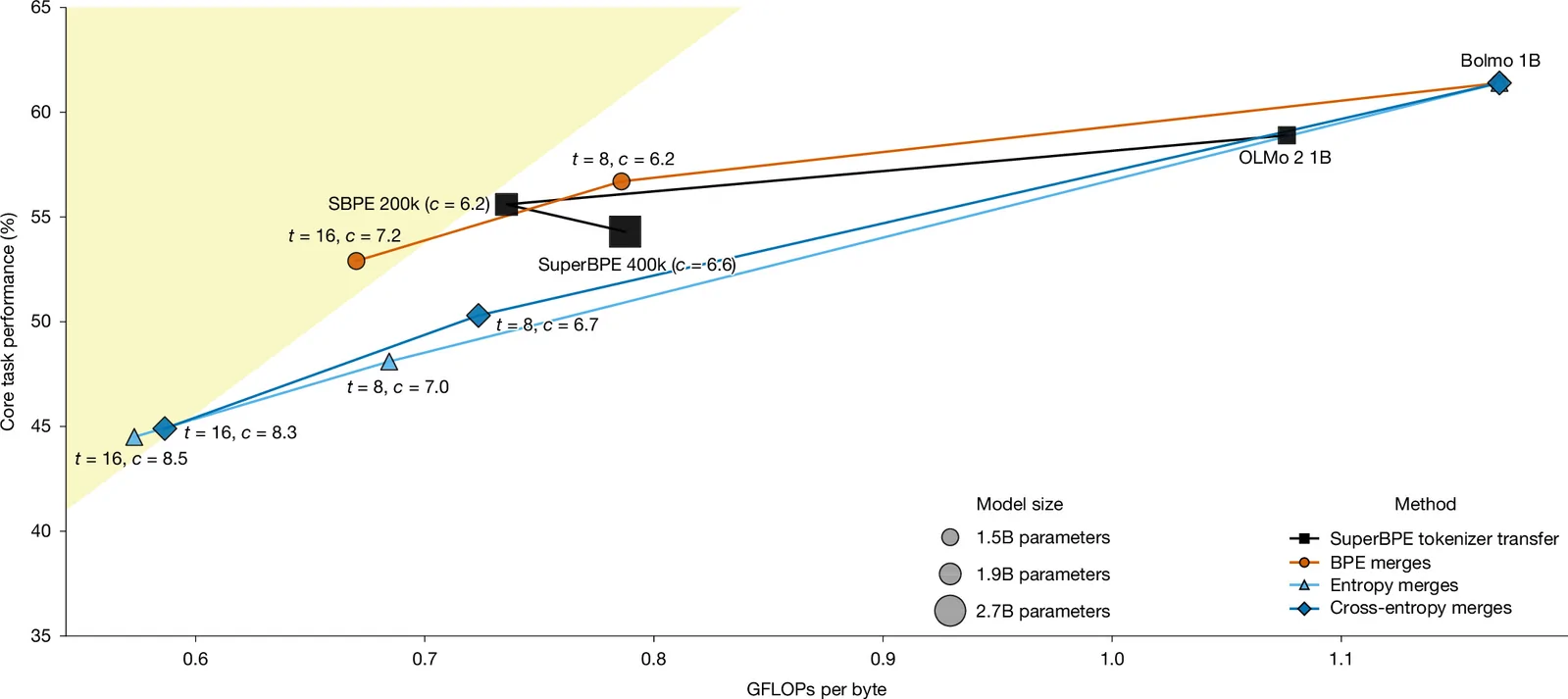
Task performance versus efficiency Pareto frontier. Results are shown for the source subword-level LLM with tokenizer transfer to SuperBPE and for Bolmo models with the boundary prediction adapted to achieve higher compression (section ‘Increasing model efficiency’). The subword-level LLM becomes Pareto-dominated as the cost of the softmax starts to become more computationally expensive for larger vocabulary sizes. Byte-level LLMs become Pareto-optimal at that point. The shaded region illustrates the optimal performance–efficiency trade-off. Pareto- *t* and *c* denote the target compression and the attained compression, respectively. Error bars indicate a 95% confidence interval of the core task macro average from binomial sampling error (*n* = 53,879 evaluation instances); each point is a single 150,000-step training run.

Our results are shown in Fig. 2. Through a transfer to SuperBPE, we could speed up the subword-level LLM while retaining performance to a large extent. However, at some vocabulary size threshold, the subword-level LLM becomes Pareto-dominated as the softmax becomes more computationally expensive. For OLMo 2 1B this happens at a vocabulary size between 200k and 400k tokens; although softmax factorizations^40,41^ would potentially avoid this, they have been observed to lead to slower convergence and increased training instability^21^ and would require a new embedding initialization methodology. Byte-level LLMs do not suffer from the softmax bottleneck. This would enable an unbounded increase in efficiency for a smooth drop-off in performance.

Interestingly, BPE merges outperformed entropy and cross-entropy merges, in contrast with earlier work using entropy-based patch boundaries^22,24^. We believe that this may be a pattern specific to the byteifying setting, as the BPE merging strategy makes the fewest distinct merges to achieve any target compression (and thus, in this sense, it is closest to the pretrained model).

### Post-training byteified models through task arithmetic

**Fig. 3:**
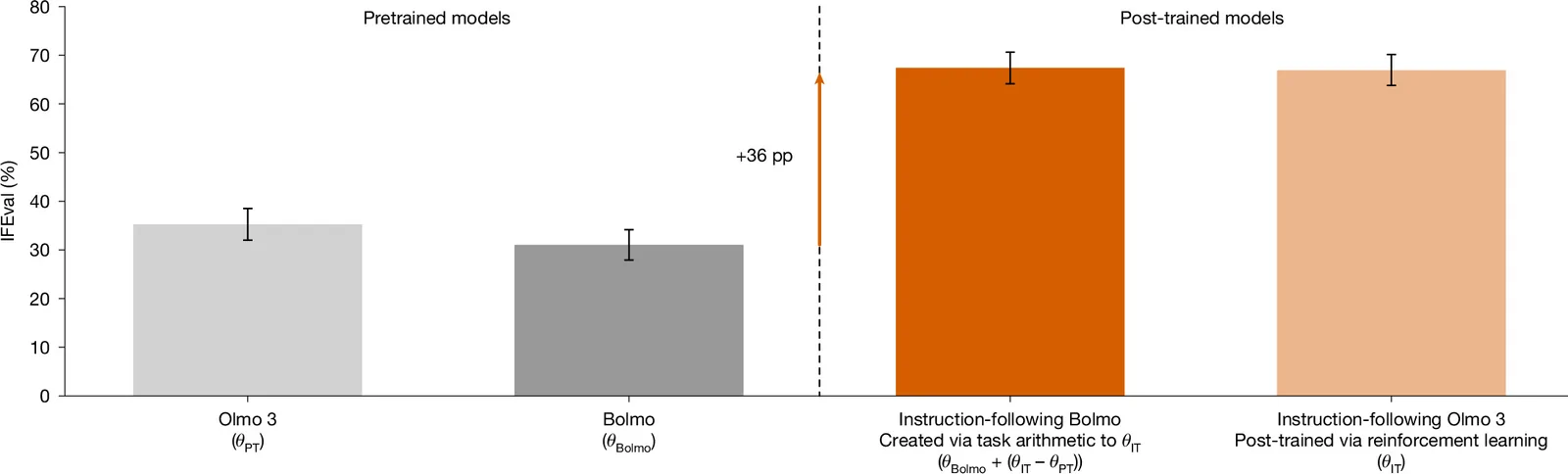
Byteified models can be post-trained by leveraging an existing subword-level post-trained model. Shown is the mean instruction following accuracy on the IFEval benchmark^45^ for the base Olmo 3 (*θ*_PT_), Bolmo (*θ*_Bolmo_), a post-trained Olmo 3 checkpoint (*θ*_IT_) and the result of merging the post-trained checkpoint into Bolmo, creating an instruction-following Bolmo. Error bars indicate a 95% confidence interval around the mean over 10,000 bootstrap resamples (*n* = 834). pp, percentage points.

Byteification adds a new component (a byteified model) to the ecosystem around the source LLM. A natural question is: How does this new component interact with the other components of the source LLM ecosystem? We, thus, investigated whether we could merge existing post-trained versions of Olmo 3 to post-train Bolmo without any extra training cost. We used the Olmo 3 checkpoint directly post-trained on following instructions through reinforcement learning (called RL-Zero^28^) as a case study. We found that we can infuse the instruction-following capabilities from this checkpoint into Bolmo with task arithmetic^42^ by adding the weight difference between the transformer layers of the post-trained checkpoint and the base Olmo 3 to the corresponding Bolmo layers (Fig. 3).

Although the Bolmo base model originally performed worse than Olmo 3 on IFEval, merging with task arithmetic lifted its performance to on par with the original post-trained checkpoint. We conclude that it is possible to use components of the subword-level LLM ecosystem to improve the corresponding byteified model. This removes the prerequisite for byte-level LLM support in the infrastructure that subword-level LLM post-training has benefitted immensely from^43,44^ and substantially speeds up iteration times.

**Extended Data Fig. 2:**
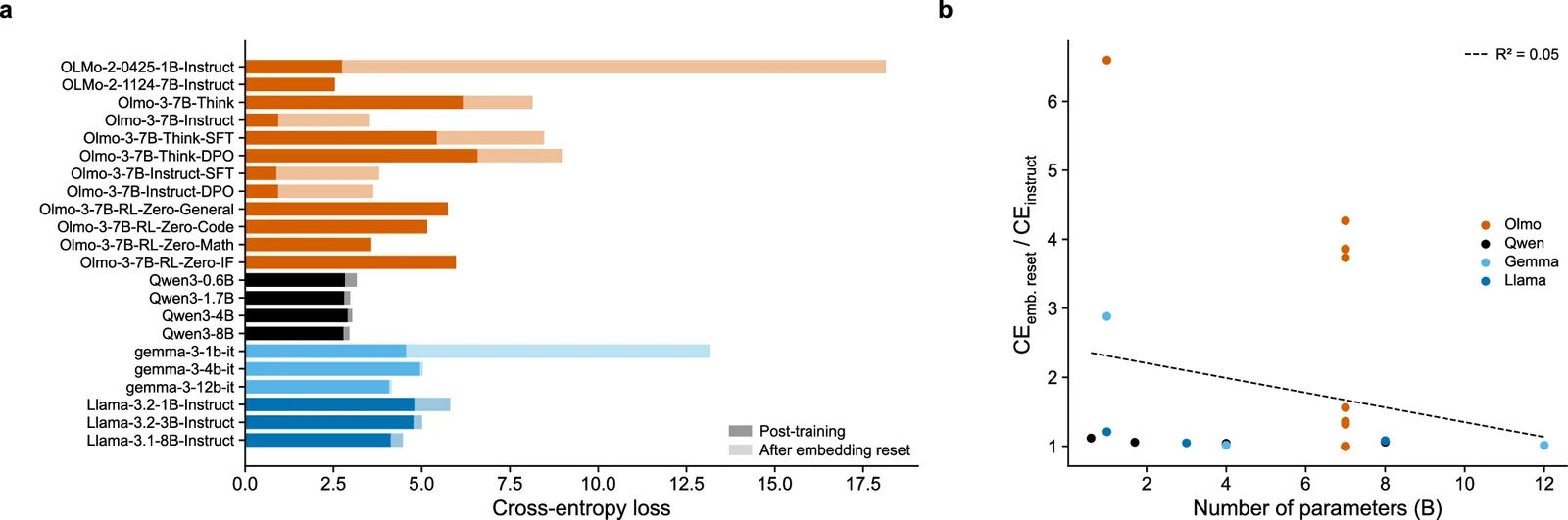
Embedding resettability across models. Since we only have a one-to-one correspondence between the parameters of the source subword-level LLM and the parameters of the global model ${\mathcal{M}}$, we can only easily adapt ${\mathcal{M}}$ via task arithmetic. The local encoder ${\mathcal{E}}$ and decoder ${\mathcal{D}}$ remain in the base model space. Whether the post-training transfer is successful thus depends on whether the base input embedding space and the base output embedding space remain compatible with post-trained inner Transformer layers; that is, on whether *resetting the embeddings of the post-trained model to the base model embeddings preserves performance*. **a**, Cross-entropy loss for post-trained models, and the same post-trained models with the embeddings reset to the corresponding base model embeddings; loss is computed on examples from the Tulu 3 dataset^43^. **b**, Number of model parameters vs. the loss ratio of the model with reset embeddings to the original post-trained model. The number of parameters explains some variance (with larger models being more amenable to reset embeddings), and models post-trained via reinforcement learning (the Olmo 3 RL-Zero family^28^) are more amenable to embedding resetting; this is in line with the findings of Shenfeld et al.^102^.

A subtle requirement when post-training byteified models through task arithmetic is that the embedding values of the post-trained models need to be resettable to the values of the base model embeddings without a large performance loss, which is generally, but not always, the case (Extended Data Fig. 2).

### Benefits of training in two stages

Training in two stages adds implementation complexity. We ran experiments where we immediately started from stage 2 to test whether the extra complexity was warranted. A fair comparison of stage 2 only training with stage 1 + stage 2 training is difficult: stage 1 training required less computation (measured in floating-point operations or FLOPs), as we only backpropagated through a fraction of the global model (‘Byteifying procedure’ in Methods), and was more memory efficient as we needed to store only a small fraction of the optimizer states. We accounted for this difference by approximately matching the FLOPs and disregarding the memory mismatch. Stage 1 needed approximately $2\times {\mathrm{FLOPs}}_{{\mathcal{M}}}$, whereas stage 2 needed approximately $3\times {\mathrm{FLOPs}}_{{\mathcal{M}}}$ (×1 for the forward and ×2 for the backward pass through the global model). We, thus, added 9.8B × 2/3 = 6.5B tokens to stage 2 training when omitting stage 1 (increasing the length of stage 2 by 17%). In practice, we believe the factor of 2/3 may have slightly favoured the stage 2 only run as the memory requirements for stage 1 were lower and inference-specific optimizations can be used to speed up the forward pass of the subword-level LLM used in stage 1.

**Fig. 4:**
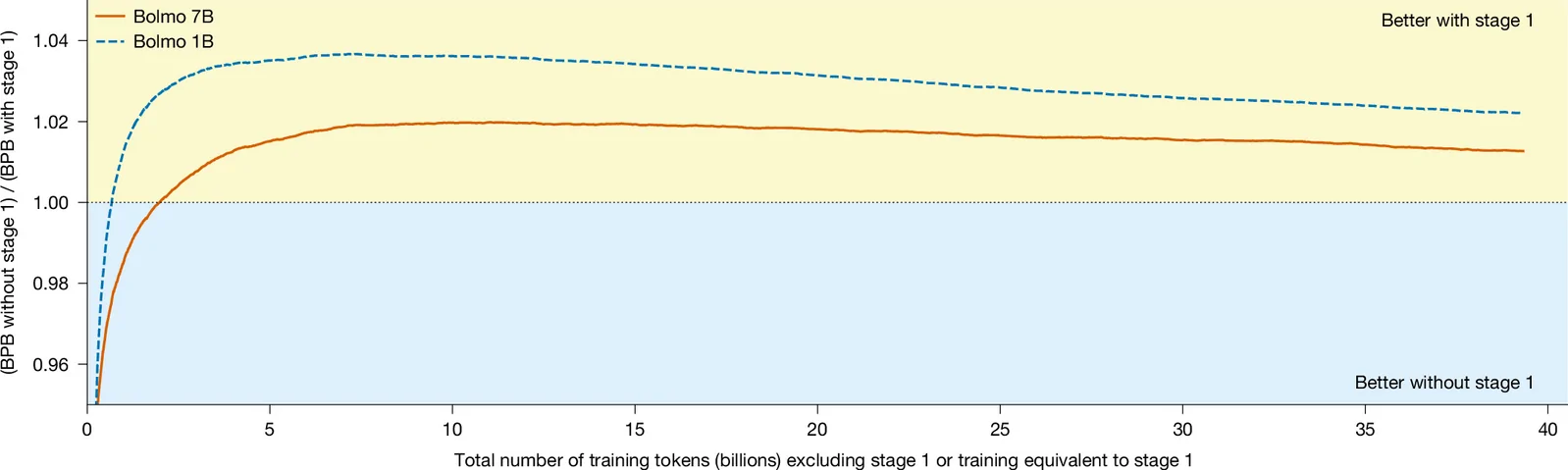
Ablating the importance of stage 1. Shown are ratios of bits per byte (BPB) throughout training of runs without stage 1 to runs with stage 1. For runs with stage 1, we excluded the stage 1 loss trajectory. For runs without stage 1, we excluded the first 9.8B × 2/3 = 6.5B tokens, resulting in a comparable trajectory over the remaining 39.3B tokens. A ratio of over 1 implies that stage 1 is beneficial. Heavy lines are exponential moving averages over 20,000 steps; faint lines, the same ratio at a 200-step average, showing the batch-to-batch variability.

Figure 4 compares the training trajectory of runs with versus without stage 1 training. The 1B model benefitted more from stage 1 training than 7B, indicating that larger models may be more robust to catastrophic forgetting when starting directly with stage 2. The bits-per-byte gap narrowed throughout the training trajectory but remained in favour of adding stage 1. As the absence of stage 1 did not cause a catastrophic degradation, we believe it is a reasonable hypothesis that stage 1 training becomes less important with larger token budgets; however, this might be influenced in non-trivial ways by factors such as the choice of data mix. A key benefit of stage 1 is streamlining experimentation: quickly obtaining a checkpoint that should come close to the performance of the subword-level LLM creates a substantially shorter feedback loop than repeatedly running full training experiments.

### Conclusion

We have established byteification as a missing direction for training from random initialization when developing byte-level LLMs. Byteification lets us train byte-level LLMs with a performance close to that of state-of-the-art subword-level LLMs at the 1B and 7B parameter scales. This is enabled by architectural and training decisions specifically designed for byteifying. The byteified models also come close to matching subword-level LLMs in inference speed. We also explored the increased flexibility of the byte-level models, such as arbitrarily decreasing token granularity for faster inference. Byteifying also lets us leverage other components of the ecosystem around the source subword model by byteifying post-trained models in zero-shot mode once the corresponding base model has been byteified. Overall, byteifying could make byte-level LLMs a practical choice with performance close to the level of subword-level LLMs at a small fraction of the resource investment of earlier work. They may enable future research directions for both the byteification setting and training from random initialization of byte-level LLMs (‘Future directions’ in Supplementary Information).

## Methods

### Byteified model architecture details

Our architecture follows the same overall structure as earlier LTLMs, including DTP, BLT and H-Net (‘Related work’ in Methods). It consists of the following components.

#### Tokenization and embedding

${\mathcal{T}}$ assigns every input UTF-8 byte in *x* (we treat *x* as a sequence over bytes: *x* ∈ {0, …, 255}^*n*^) a corresponding embedding in ${{\mathbb{R}}}^{d}$ from an embedding table containing an entry for every byte. The embedding table over bytes is negligible in size compared with embedding tables over subwords. However, scaling the size and sparsity of the embedding table has been shown to improve performance while having no negative effect on inference speed^46^. Inspired by the hash embeddings in BLT^22,47^, we, thus, increased the size of the embedding table. Specifically, we residually added the longest subword embedding (from the original embedding table in the subword-level LLM) that ends at the current byte position to every byte embedding:

$${e}_{i}:= {{\mathcal{T}}}_{\mathrm{Byte}}({x}_{i})+{{\mathcal{T}}}_{\mathrm{SubwordSuffix}}({x}_{:i}),$$

where ${{\mathcal{T}}}_{\mathrm{SubwordSuffix}}$ assigns an embedding to every byte based on the index of the subword token in the vocabulary ${{\mathcal{V}}}_{\mathrm{Subword}}$ with the longest common suffix to the byte sequence up to the current position *i*. For example, the subword embeddings corresponding to {‘_’, ‘_f’, ‘_fl’, ‘o’, ‘_flow’} could be added to the byte representation of the sequence {‘_’, ‘f’, ‘l’, ‘o’, ‘w’}, assuming none of {‘_flo’, ‘flo’, ‘lo’} are in the original subword vocabulary. Retaining the subword embeddings is not strictly necessary, and we can generally achieve the same performance by increasing the size of the local encoder instead. However, retaining the subword embedding allows us to achieve a better performance–efficiency trade-off by increasing the number of cheap sparsely activated parameters; an alternative to increasing the size and sparsity of the local encoder is using a mixture of experts in the feedforward layer, although we do not investigate this here.

#### Local encoder

**Extended Data Fig. 3:**
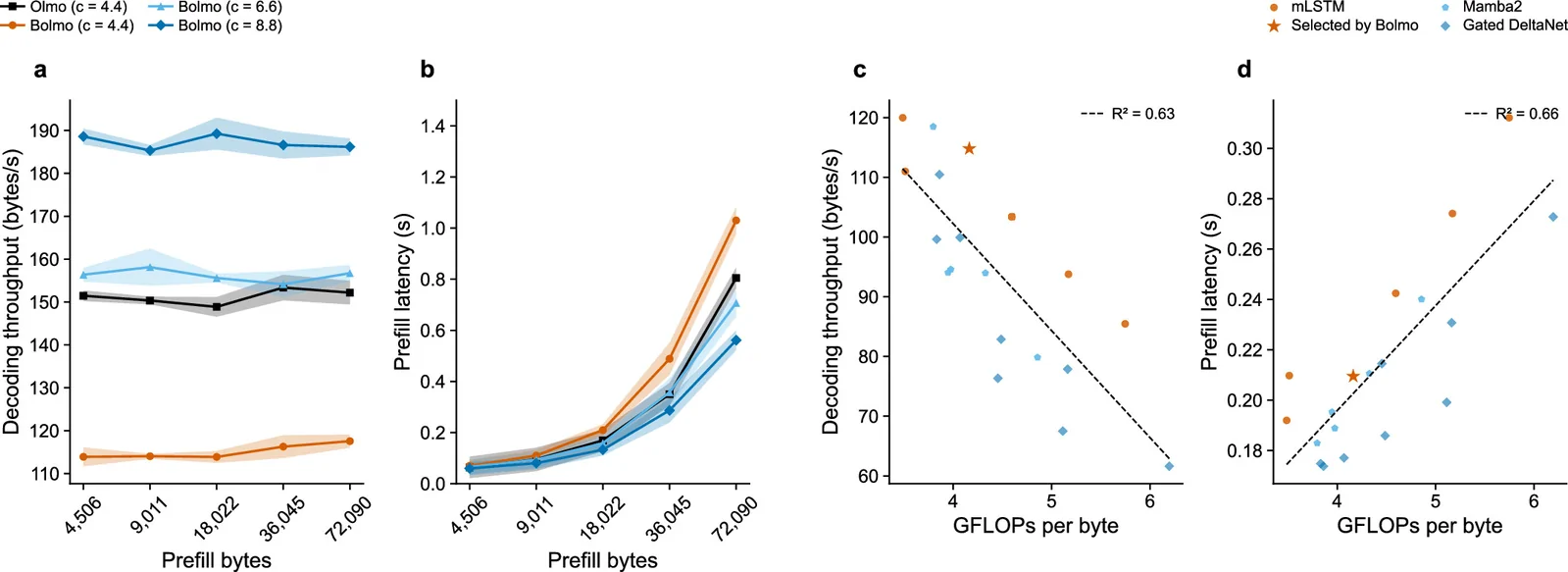
Inference efficiency measurements. **a**, Greedy decoding throughput (bytes/s) across compression factors and prefill lengths. **b**, Prefilling latency (time to first byte) across compression factors and prefill lengths; Bolmo 7B overtakes Olmo 3 7B at a compression of ~6.6 bytes per patch. **c**, Decoding throughput for 18.0K prefill bytes across candidate local model architectures and of the final chosen architecture. **d**, Prefilling latency for the same candidate models and the final chosen architecture. All numbers recorded at batchsize  =  1 on H100 GPUs. Non-greedy sampling affects throughputs by <  5% in our experiments. Shaded areas indicate one standard deviation.

The local encoder ${\mathcal{E}}$ contextualizes the byte-level embeddings through an mLSTM layer^33^, resulting in the contextualized representations $\widehat{e}$. We found that mLSTM improves inference speed compared with other linear recurrent neural network (RNN) variants (Extended Data Fig. 3) while attaining competitive performance. We found a single mLSTM layer to be sufficient, as the expressivity of the local encoder was substantially enhanced by the retained subword embeddings.

#### Boundary predictor

The boundary predictor ${\mathcal{B}}$ predicts a score *p* ∈ [0, 1] for each byte based on the contextualized representations $\widehat{e}$. If *p* is greater than some threshold, a patch boundary is placed after the current byte. In contrast to earlier LTLMs, the boundary predictor of the byteified models is non-causal: it has access to 1 byte of future context, and it is only employed for the prefill, where future information can be used while retaining the ability to generate text. We describe non-causal boundary prediction in detail in section ‘Byteified language model architecture’, where we also discuss how boundary prediction is handled during decoding.

#### Pooling

We pool byte-level representations into patch representations by selecting the representation of the last byte in every patch as the patch-level representation *h*. This is equivalent to the pooling done by Hwang et al.^2^ and does not introduce any extra parameters. Unlike Hwang et al.^2^, the local models and the global model use the same representation dimensionality, obviating the need for an up-projection. We originally experimented with smaller local dimensions but found the up-projection mechanism to bottleneck performance by restricting the rank of the representations (Extended Data Fig. 1).

#### Global model

Most of the compute is spent in the deep global model ${\mathcal{M}}$ contextualizing the patch representations *h* into $\widehat{h}$. We retain the global model of the original subword-level LLM.

#### Depooling

The global model is invoked at every patch boundary, providing contextualized representations for every patch. It remains to depool these representations back to representations of bytes. We do so by adding the latest available patch representation in $\widehat{h}$ at any byte position to a linear projection of the byte representations $\widehat{e}$, resulting in *z*. This is like the depooling by H-Net, again forgoing the projection due to equal global and local dimensionality.

#### Local decoder

The local decoder ${\mathcal{D}}$ contextualizes the depooled byte representations *z* into $\widehat{z}$ through another stack of mLSTM layers. We used more mLSTM layers (in practice, four) to increase capacity, because unlike in the encoder, we found it infeasible to meaningfully reincorporate the output subword embedding matrix, which could have potentially allowed us to reduce the number of layers in the decoder in a similar way as for the encoder.

#### Language-modelling head

The language-modelling head converts the final byte representations $\widehat{z}$ into scores interpretable as next-byte probabilities (and next-byte-followed-by-boundary probabilities if boundary predictions are fused; compare with ‘Byteified language model architecture’) through a projection to the vocabulary space and softmax. During decoding, the next atom (byte or boundary in the unfused case and byte or byte-and-boundary in the fused case) was generated by looping over the local encoder, local decoder and language-modelling head. If a boundary is predicted, the patch ends and is passed through the global model, after which the next loop over the local encoder, local decoder and language-modelling head starts. We did not find it necessary to employ any mechanism to ensure consistency between the patch representations and decoded bytes. As in standard subword-level language models, the extent of the possible mismatch between the hidden representations and the decoded atoms is dictated by the sampling procedure; any apparent mismatch is analogous to sampling a non-argmax token in a standard subword-level language model.

In practice, Bolmo 1B contains approximately 10M fewer parameters, where M is millions, than OLMo 2 1B (−0.7%), Bolmo 7B contains approximately 330M more than Olmo 3 7B (+4.5%), Blama 8B contains approximately 220M more than Llama 3 8B (+2.7%) and Bwen 8B contains approximately 120M more than Qwen3 8B (+1.5%).

### Byteifying procedure

#### Subword-to-byte distillation

The first stage starts by initializing the parameters of the global model from the subword-level LLM checkpoint, whereas the parameters of the local models and the language-modelling head are initialized randomly. The aim of this stage is to quickly learn weights for the local encoder, local decoder, boundary predictor and language-modelling head that recover the behaviour of the subword model. Efficiency is crucial; the cost of this stage should be minimal to permit fast experimentation and allow increasing the investment into stage 2. To achieve these goals, we designed a stage 1 procedure that allows the model to learn the desired weights without fully backpropagating through the global model. This substantially reduces the time per training step (Extended Data Table 4). The stage 1 loss is minimal if and only if the byte-level model exactly mimics the source subword-level LLM. It is composed of three parts.

##### Quickly learning a boundary predictor ${\boldsymbol{\mathcal{B}}}_{{\bf{byteify}}}$

We train the boundary predictor to emulate the boundaries placed by subword tokenization through a binary cross-entropy loss:

$${{\mathcal{L}}}_{{\mathcal{B}}}:= -\sum _{t}({{\mathcal{B}}}_{\mathrm{subword}}{(x)}_{t}\,\log \,{{\mathcal{B}}}_{\mathrm{byteify}}{(\hat{e})}_{t}+(1-{{\mathcal{B}}}_{\mathrm{subword}}{(x)}_{t})\,\log (1-{{\mathcal{B}}}_{\mathrm{byteify}}{(\hat{e})}_{t})),$$

where ${{\mathcal{B}}}_{\mathrm{subword}}(x)$ is 1 for every byte at the last position of a subword patch, otherwise 0. The boundary predictor ${{\mathcal{B}}}_{\mathrm{byteify}}$, which uses future context to tokenize the prefill text, quickly achieves over 99% accuracy.

##### Quickly learning a local encoder $\boldsymbol{\mathcal{E}}$

Assuming our boundary predictor perfectly emulates subword tokenization, our local encoder and pooling mechanism will be a perfect substitute for the subword embedding matrix if they yield the same input to the global model as the subword embedding matrix for every patch. This is the case if all pooled representations $\mathrm{Pool}({\mathcal{E}}({\mathcal{T}}\,(x)),{{\mathcal{B}}}_{\mathrm{byteify}}(\hat{e}))$ are equal to the corresponding subword embeddings ${{\mathcal{T}}}_{\mathrm{subword}}(x)$. Hwang et al.^2^ optimized towards this goal by directly minimizing the *L*^2^ distance of every pooled representation to the corresponding subword embedding. We took an alternative approach inspired by research on model stitching, which showed that similar representations do not necessarily propagate through subsequent layers in a similar way^48^. We propagate the pooled representations through *n* layers of the global model and minimize the *L*^2^ distance to the subword representations that result from propagating the subword embeddings through the same *n* layers:

$$\begin{array}{l}{Y}_{{\mathcal{E}}}:= {{\mathcal{M}}}_{:n}({{\mathcal{T}}}_{\mathrm{subword}}(x)),\\ {\hat{Y}}_{{\mathcal{E}}}:= {{\mathcal{M}}}_{:n}(\mathrm{Pool}({\mathcal{E}}({\mathcal{T}}\,(x)),{{\mathcal{B}}}_{\mathrm{subword}}(x))),\\ {{\mathcal{L}}}_{{\mathcal{E}}}:= \parallel {Y}_{{\mathcal{E}}}-{\hat{Y}}_{{\mathcal{E}}}\parallel .\end{array}$$

where ${Y}_{{\mathcal{E}}}$ are the representations of the original model at layer *n*, ${\hat{Y}}_{{\mathcal{E}}}$ the retrofitted model representations at the same layer *n* of the global model, and ${{\mathcal{L}}}_{{\mathcal{E}}}$ the ${{\mathcal{L}}}^{2}$ distance between the two. Notably, we pool the local encoder representations using the true subword boundaries ${{\mathcal{B}}}_{\mathrm{subword}}$ instead of ${{\mathcal{B}}}_{\mathrm{byteify}}$; this is necessary to preserve the alignment of the pooled representations to the representations in ${{\mathcal{T}}}_{\mathrm{subword}}(x)$ along the sequence dimension. ${{\mathcal{M}}}_{:n}$ indicates the global model up to and including the *n*th layer. The weights of ${\mathcal{M}}$ are kept frozen. If *n* = 0, this reduces to the setting of ref. ^2^. Although choosing *n* > 0 necessitates backpropagating through some parts of the global model, we can minimize the resulting cost by choosing a small *n*. We found that *n* = 4 strikes a good balance between performance and efficiency, as it substantially outperformed *n* = 0 while remaining cheap to compute.

##### Quickly learning a local decoder $\boldsymbol{\mathcal{D}}$

Our local decoder and language-modelling head are optimal if our byte-level LLM assigns the same likelihood as the subword model to every text *x*. Assuming equal patch boundaries, it is optimal if the likelihood of every patch is equal. As subword-level LLMs implicitly predict output patch boundaries, we cannot easily compute comparable patch likelihoods in byte-level models without output boundary prediction. In this case, we would have to resort to approximations, as in ref. ^49^. However, as the byteified models do predict output patch boundaries, simply comparing the likelihoods of every patch results in an exact objective (proof in ‘Exactness of the stage 1 objective’ in Supplementary Information):

$$\begin{array}{l}{{\mathcal{L}}}_{{\mathcal{D}},\mathrm{Distill}}:= \sum _{i}f\left(\prod _{j\in T(x,i)}\,\mathrm{LMHead}\,({\hat{z}}_{\mathrm{subword}})[\,j,\mathrm{next}\_\mathrm{byte}(x,j)]\right.\\ \,\left.\parallel \,{\mathrm{LMHead}}_{\mathrm{subword}}({z}_{\mathrm{subword}})[i,\mathrm{next}\_\mathrm{tok}(x,i)]\right),\end{array}$$

where *j* ∈ *T*(*x*, *i*) indicates all byte indices *j* that are part of the *i*th subword patch; this includes the indices of the special <b> symbol if treated as separate or the indices of the 256 special symbols consisting of a byte plus <b> if fused. next_tok( ⋅ ) and next_byte( ⋅ ) map to the index in the vocabulary of the symbol occurring after the current symbol (token or byte), including special symbols. ${z}_{\mathrm{subword}}={\mathcal{M}}({{\mathcal{T}}}_{\mathrm{subword}}(x))$ are the representations of the subword model at the final layer, ${\hat{z}}_{\mathrm{subword}}={\mathcal{D}}(\mathrm{Depool}\,(\hat{e},{z}_{\mathrm{subword}},p))$ is the result of passing these representations through the depooling layer and the local decoder, and LMHead_subword_ is the language-modelling head of the source subword-level LLM. We chose for the comparison function *f*:

$$f(\hat{y}\,\parallel \,y):= -({y}^{1/\tau }\,\log \,{\hat{y}}^{1/\tau }+(1-{y}^{1/\tau })\,\log (1-{\hat{y}}^{1/\tau })),$$

where $\widehat{y}$ are the predictions and *y* the targets. In principle, *f* could be any function that is minimal at $\widehat{y}=y$; we chose the temperature-modulated binary cross-entropy with temperature *τ* = 5, as recommended by Minixhofer et al.^49^. In practice, we conducted the operations involved in the loss computation in log-space to ensure stable numerics. We optionally combine the distillation loss ${{\mathcal{L}}}_{{\mathcal{D}},\mathrm{Distill}}$ with a cross-entropy loss to encourage the system to model the training data well and to start exploiting byte-level information, at the cost of giving up exactness if enabled:

$${{\mathcal{L}}}_{{\mathcal{D}},\mathrm{CE}}:= \sum _{j}-\log \,\mathrm{LMHead}\,({\hat{z}}_{\mathrm{subword}})[\,j,\mathrm{next}\_\mathrm{byte}(x,j)].$$

##### Putting it together

In principle, the boundary predictor and local encoder on the one hand and the local decoder and language-modelling head on the other could be trained separately (assuming we stop the gradient to the encoder through ${\widehat{z}}_{{\rm{subword}}}$). Although there may be scenarios where this is beneficial, we chose to train them together for simplicity. The complete stage 1 loss is given by

$${{\mathcal{L}}}_{\mathrm{Stage1}}:= {\lambda }_{{\mathcal{B}}}{{\mathcal{L}}}_{{\mathcal{B}}}+{\lambda }_{{\mathcal{E}}}{{\mathcal{L}}}_{{\mathcal{E}}}+{\lambda }_{{\mathcal{D}},\mathrm{Distill}}{{\mathcal{L}}}_{{\mathcal{D}},\mathrm{Distill}}+{\lambda }_{{\mathcal{D}},\mathrm{CE}}{{\mathcal{L}}}_{{\mathcal{D}},\mathrm{CE}},$$

where ${\lambda }_{{\mathcal{B}}},{\lambda }_{{\mathcal{E}}},{\lambda }_{{\mathcal{D}},\mathrm{Distill}},{\lambda }_{{\mathcal{D}},\mathrm{CE}}\in {\rm{{\mathbb{R}}}}$ are the loss weights, which we set as follows: ${\lambda }_{{\mathcal{B}}}=4,{\lambda }_{{\mathcal{E}}}=1,{\lambda }_{{\mathcal{D}},\mathrm{Distill}}=1\,\mathrm{and}\,{\lambda }_{{\mathcal{D}},\mathrm{CE}}=1$. Stage 1 needs in total one forward pass through all layers and one backward pass through the first *n* layers of the global model plus forward and backward passes through local encoder, local decoder, boundary predictor and language-modelling head. This makes stage 1 substantially more efficient than training the entire model. It could also be further optimized by quantizing or applying inference-specific optimizations to the global model layers starting from the (*n* + 1)th layer (which we do not need to backpropagate through). We analysed the difference between inserting stage 1 and directly training the entire model end-to-end with randomly initialized parameters (besides the global model) in ‘Benefits of training in two stages’. Besides performance improvements, stage 1 provides a vehicle for rapid experimentation: we can conduct stage 1 training to rapidly check whether a particular architecture for the local encoder and decoder has sufficient capacity to emulate the input and output embedding matrices, respectively. We use this to guide the architecture search for our byteified models under the hypothesis that byte-level architectures that cannot emulate the subword model after stage 1 will remain inadequate with further stage 2 training.

#### End-to-end training

In the second stage, we train the entire model end-to-end, retaining only the boundary loss ${{\mathcal{L}}}_{{\mathcal{B}}}$ and the cross-entropy loss. For the cross-entropy loss (previously denoted ${{\mathcal{L}}}_{{\mathcal{D}},\mathrm{CE}}$), we substitute the depooled representations ${\widehat{z}}_{{\rm{subword}}}$ computed from the subword model representations with the true depooled representations $\widehat{z}$, and we refer to this new loss as ${{\mathcal{L}}}_{\mathrm{CE}}$ instead:

$${{\mathcal{L}}}_{\mathrm{Stage2}}:= {\lambda }_{{\mathcal{B}}}{{\mathcal{L}}}_{{\mathcal{B}}}+{\lambda }_{\mathrm{CE}}{{\mathcal{L}}}_{\mathrm{CE}}.$$

We now optimize all parameters, including those of the global model ${\mathcal{M}}$. This stage is intended for the model to adjust to the end-to-end setting, as in stage 1 we assumed a local encoder and boundary predictor perfectly emulating the subword model, which, although close, is not true in practice. The global model learns to exploit the new byte-level information in stage 2 and can optionally be trained with higher compression ratios of bytes per patch (section ‘Increasing model efficiency’).

#### Methods for increasing the compression factor

To increase the compression factor of a byteified model, we fix a compression ratio *t* for the target average bytes per patch. We then remove subword boundaries (merged subword tokens) of ${{\mathcal{B}}}_{\mathrm{subword}}$ until the desired compression ratio is achieved. We experimented with three merging strategies:

1. BPE. We iteratively merged the most common pair of tokens as in byte pair encoding^1^. In contrast to conventional BPE, we applied BPE per example instead of over the entire corpus. This was inspired by Feher et al.^50^, who showed that it is possible to retrofit language models to operate over BPE merges of the tokens in their vocabulary.
2. Entropy. We used an auxiliary 370M-parameter subword-level LLM, trained on 74.3B tokens following a downscaled version of the OLMo 2 training and architecture^29^, to compute next-token entropies. We then iteratively merged the pair of patches that, when summing their individual entropies, resulted in the lowest entropy among all entropy sums of pairs of patches in the example.
3. Cross-entropy. We used the same auxiliary LLM as for entropy-based merging, but instead of merging the pair of tokens with the lowest total entropy, we iteratively merged the pair of tokens with the lowest total cross-entropy with respect to the next token in the data.

For entropy- and cross-entropy-based merging, the auxiliary LLM was required during training time only to supervise the boundary predictor (as in DTP^24^). Unlike BLT^22^, we did not need to retain the auxiliary LLM for inference.

Even though the loss is discontinuous with respect to the parameters of the boundary predictor and we did not employ any technique to backpropagate through the discrete boundary predictions, we observed stable training without loss spikes with all of the above merging methods. An important nuance is that the supervision target compression ratio *t* was not attained by the model. Despite the boundaries not being learned end to end, the model learns to trade off boundary prediction accuracy with the main next-byte prediction loss, like other multitask models that learn to balance performance on the constituent tasks (for example, ref. ^51^). An important hyperparameter is, thus, the factor ${\lambda }_{{\mathcal{B}}}$ controlling the importance of the boundary prediction task; we keep ${\lambda }_{{\mathcal{B}}}=4$ from stage 1 training.

### Experimental set-up

#### Data

Our data mix consists of approximately 172B tokens, as tokenized by the Dolma2 Tokenizer (https://huggingface.co/allenai/dolma2-tokenizer) from the Dolma 3 pretraining data mix^28^, augmented with 75M tokens of CUTE-style data^11^, sampled so as not to overlap with the CUTE test set, to encourage character understanding (‘Character-level training data’ in Methods). Training ran for less than one epoch on this mix.

#### Model

We used Qwen3 8B Base^30^, Llama 3 8B^31^ and the Olmo 3 7B checkpoint after mid-training and long-context extension^28^ as the starting points for our byteified models. For the local models, we used stacks of alternating mLSTM^33^ and feedforward layers of size 1 and 4 for the encoder and decoder, respectively (Extended Data Table 5).

#### Training

We trained stage 1 on 9.8B tokens (approximately 43B bytes). In this stage, we trained the local encoder, decoder, boundary predictor and language-modelling head, keeping the global model frozen. For stage 2, we trained the entire model on 39.3B tokens (approximately 173B bytes; Extended Data Table 4).

#### Evaluation

We created the 7B byteification evaluation suite based on the Olmo 3 OlmoBaseEval^28^, skipping GSM Symbolic and BigCodeBench due to their size, and adding CUTE^11^ and EXECUTE^52^ to measure character understanding in English and across other languages, respectively. We created the 1B byteification evaluation suite based on the Base Easy Suite in Olmo 3^28^, again adding CUTE^11^ to measure character understanding. For the 1B suite, we defined a set of core tasks consisting of ARC^53^, MMLU^54^, CSQA^55^, HellaSwag^56^, WinoGrande^57^, SocialIQA^58^, PiQA^59^, the Basic Skills benchmark^28^ and CUTE^11^ for use in ablations and sweeps (Extended Data Tables 1 and 2). The 7B suite additionally comprises HumanEval^60^, MBPP^61^, DS-1000 (ref. ^62^), DeepSeek LeetCode^63^, MultiPL-E^64^, GSM8K^65^, Minerva MATH^66^, MedMCQA^67^, MedQA^68^, SciQ^69^, DROP^70^, Natural Questions^71^, SQuAD^72^, CoQA^73^ and Lambada^74^, in addition to the Jeopardy and Basic Skills tasks from OlmoBaseEval^28^. We used PyTorch v.2.8 (ref. ^75^) and vLLM v.0.11.0 (ref. ^76^) to generate completions whenever available. Generation capabilities for the byteified models are implemented in our bolmo-core (https://github.com/allenai/bolmo-core) framework.

### Character-level training data

To encourage models trained on our data mix to learn information about the characters within a word, we generated approximately 75M tokens (approximately 0.04% of the training data) for tasks requiring character-level understanding using the CUTE repository (https://github.com/Leukas/CUTE). Tasks included spelling out words, reversing words as well as swapping, deleting and substituting characters within a word given words in a source wordlist. We used a list of *n* = 150,000 words, ensuring zero overlap with the CUTE test words to avoid contamination. These data are purely in English. We did not use any multilingual character-understanding data, but we still observed large improvements on the multilingual EXECUTE benchmark, indicating that some texts requiring character-level understanding can help acquire generalizable knowledge about the characters within words. We observed that byte-level models otherwise do not acquire this knowledge through our short training schedule. However, training for longer, on more diverse data or with larger local models could act as alternative routes to acquiring character-level knowledge.

### Statistical tests

To test whether model A statistically significantly outperforms model B, we started from the set of benchmark scores, *D* = {(*a*_1_, *b*_1_), (*a*_2_, *b*_2_), …, (*a*_*n*_, *b*_*n*_)}, where (*a*_*i*_, *b*_*i*_) are the scores of model A and model B on benchmark *i*. The set of benchmarks consists of all *n* tasks in the respective evaluation suite (the 7B byteification suite or 1B byteification suite with *n* = 40 and *n* = 22, respectively; Extended Data Tables 1 and 2). We then drew *n* pairs from *D* with replacement for every bootstrap iteration *k* ∈ {1, 2, …, *N*} with the number of bootstrap iterations *N* = 10,000:

$${D}_{k}^{\star }=\{({a}_{{j}_{1}},{b}_{{j}_{1}}),({a}_{{j}_{2}},{b}_{{j}_{2}}),\ldots ,({a}_{{j}_{n}},{b}_{{j}_{n}})\}.$$

We then computed the difference in performance for each bootstrap sample,

$${\delta }_{k}^{\star }=\frac{1}{n}\sum _{(a,b)\in {D}_{k}^{\star }}a-b,$$

resulting in an empirical distribution of performance differences $\varDelta =\{{\delta }_{1}^{\star },{\delta }_{2}^{\star },\ldots ,{\delta }_{N}^{\star }\}$, which we used to compute an unadjusted *P* value:

$${p}^{{\rm{unadj}}}=\frac{1}{N}\mathop{\sum }\limits_{k=1}^{N}{\mathbb{1}}\,({\delta }_{k}^{\star }\le 0).$$

We then applied a Holm–Bonferroni correction for multiple comparisons. Given a total of *K* unadjusted *P* values {*P*^unadj^(*A*, *B*), *P*^unadj^(*A*, *C*), …, *P*^unadj^(*A*, *Z*)}, we sorted the *P* values in ascending order and adjusted starting from *i* = 1 (the smallest *P* value), sequentially moving to larger *P* values:

$${p}_{(i)}^{{\rm{adj}}}=\min (\mathop{\max }\limits_{j\le i}((K-j+1){p}_{(j)}^{{\rm{unadj}}}),1).$$

Finally, we undid the sorting operation to arrive at an adjusted *P* value for every comparison.

### Related work

#### Tokenization

LLMs process information represented as a discrete sequence of symbols called tokens or patches. The process of segmenting the input into this discrete sequence is called tokenization, with different ways to tokenize being used across modalities such as text^10^, audio^77^ and images^78^. The predominant approach for tokenizing text since the inception of LLMs has been subword tokenization^1,10^: tokenizing text into a discrete sequence of units from a finite vocabulary of subword tokens (usually of size 30k–300k), typically represented as integer IDs. Subword tokenization causes several problems. (1) Information about the characters within each token is lost. Although LLMs have been shown to implicitly learn the constituent characters of their tokens^11,79^ and although it is possible to explicitly re-introduce character information^12,80^, they still fall short in tasks requiring character knowledge^11,13,81^. (2) The implicit reliance of subword tokenization on the future contents of the text (called tokenization bias) causes unexpected behaviour at inference if the prompt ends in the middle of a word or with whitespace^18–20^. (3) The need for a fixed, finite subword vocabulary causes restrictive rigidity: for example, although encoding English efficiently is crucial for pretraining, as the vast majority of current pretraining documents are in English, various downstream tasks have different efficiency requirements across different languages. (4) Tokenization in contemporary LLMs is tied to compute allocation: in a standard LLM, the same amount of compute is spent on processing every token in the prefill, every token contributes equally to the key–value cache (KV cache) size and a fixed amount of compute is spent on sequentially generating any new token. Although there are ways to mitigate this problem post hoc—such as KV cache sparsification^82^ and multi-token prediction^83^—directly adapting the tokenization and, thus, the compute allocation based on the input instead might be more effective^22,24^.

#### Byte-level LLMs

The shortcomings of subword tokenization have motivated extensive work on a wide range of alternatives, which even include tokenizing text by rendering it into pixels and segmenting these into patches^84–86^. The most common alternative has been tokenizing into a smaller set of finer-grained atomic units, such as UTF-8 bytes. One strand of work directly replaces subword tokens with UTF-8 bytes, keeping other aspects of the architecture mostly the same^26,27,49,87^. This can solve problems (1) and (2) of subword tokenization and potentially problem (3) with the right choice of fine-grained units^88,89^. In any case, compute allocation remains a problem, exacerbated by having to process longer sequences. To mitigate this problem, some architectures pool a fixed number of tokens into a single representation with a lightweight local encoder (for example, another transformer network), pass the pooled representations through a deep global model operating over the shortened sequence and then depool the representations back to the original granularity through a local decoder. This approach has been pioneered for autoregressive models by the hourglass transformer^90^ and later adopted more broadly^91–93^. Recent subsequent work has shown that replacing static pooling with dynamic tokenization improves the performance–efficiency Pareto front^24,25^. In this case, the token boundaries may be learned end to end, rely on entropy spikes or be externally supervised^2,24^. We collectively refer to these architectures as LTLMs, as—although operating over bytes—they perform a tokenization step inside the model that aggregates the byte representations into representations over latent patches. Byte-level LTLMs can finally address issues (1) to (4) of subword tokenization. The most recent LTLMs have shown promise by performing on par with subword tokenization when spending the same overall number of FLOPs on training^2,22^. Although we focused on LTLMs in this work, there are also other strands of promising research relevant to byte-level models, such as MrT5^94^, which uses a soft gating mechanism to reduce sequence lengths at inference, and zip2zip^95^, which adaptively merges tokens based on the past token context.

#### Tokenizer transfer and retrofitting

Techniques to alter the architecture of a model with extra training are typically referred to as retrofitting, which often relies on self-distillation^82,96^. The principal difficulty when this involves a change of tokenizer is finding embeddings for the new tokens; this is usually done using heuristics^39,97,98^ or training-based methods^99^. Recently, effective tokenizer transfer methods based on cross-tokenizer distillation have been introduced^49,100,101^. Here the original model is seen as the teacher, the tokenizer-transferred model is seen as the student and the objective is to match the behaviour of the student to the teacher. Byteification is a special case of tokenizer transfer. Byteification was first done by Pagnoni et al.^22^ by initializing the LTLM parameters from an existing subword model where possible and training as if from random initialization. Hwang et al.^2^ later byteified by supervising the boundary prediction to match the subword boundaries and introducing an auxiliary embedding-matching loss. Our key contribution is creating an LTLM that is specifically suited to byteifying. We do so by introducing a new architecture (section ‘Byteified language model architecture’) and a dedicated two-stage procedure that efficiently byteifies by first learning to exactly recover the behaviour of the source subword model (section ‘Byteification training procedure’). Together, these innovations allow us to closely match the performance of state-of-the-art subword-level LLMs with a byteified model.

## Online content

Any methods, additional references, Nature Portfolio reporting summaries, source data, extended data, supplementary information, acknowledgements, peer review information; details of author contributions and competing interests; and statements of data and code availability are available at https://doi.org/10.1038/s41586-026-11111-4.

## Supplementary information

Supplementary Information This file contains sections: ‘Future directions’ and ‘Exactness of the stage 1 objective’ and supplementary references

Peer Review File

## Figures and Tables

**Extended Data Table 1:**
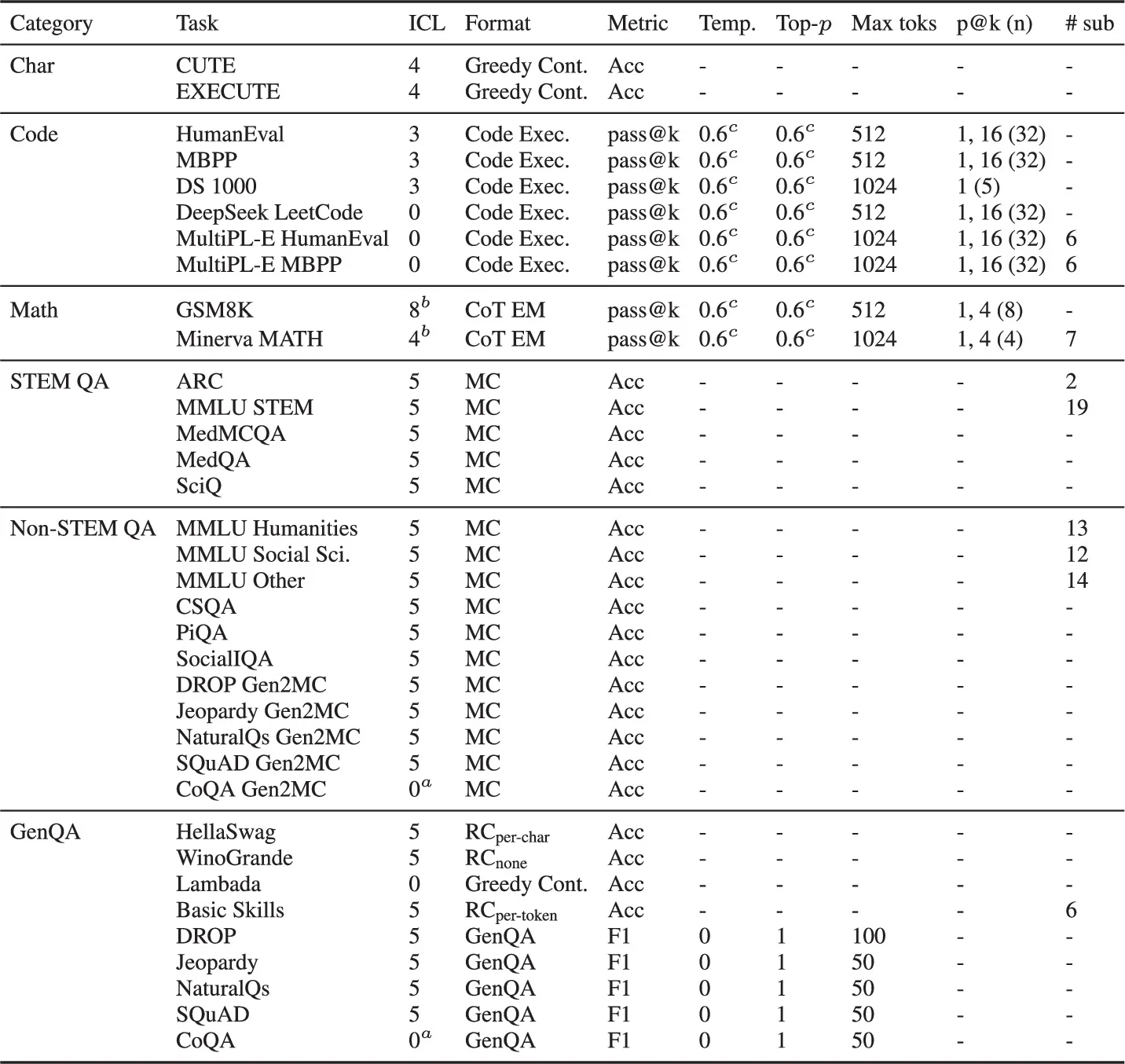
Details of the 7B byteification evaluation suite

Adapted from the evaluation suite of Olmo 3^28^. Tasks were formatted as multiple-choice (MC), rank choice (RC, following the setup of Gu et al.^103^), short-form generative (GenQA), chain-of-thought with exact-match scoring (CoT EM) or code execution (Code Exec.). ICL: number of in-context examples; Acc: accuracy; Max toks: maximum number of generated tokens; # sub: number of subtasks. ^*a*^few-shot examples are built into the task; ^*b*^human-written few-shot examples; ^*c*^temperature scaling applied before top-*p*, both applied over the concatenated 512 logits (for the 256 bytes and the 256 bytes followed by a boundary) in the case of the byteified models.

**Extended Data Table 2:**
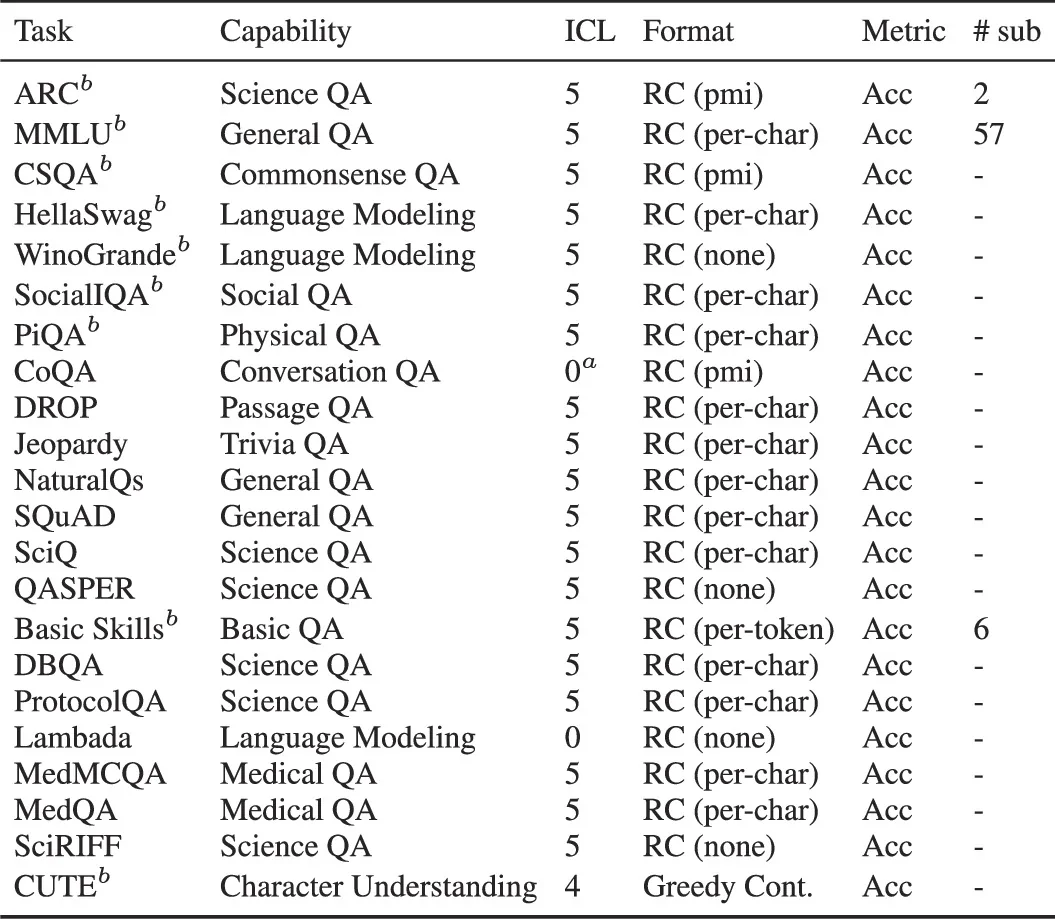
Details of the 1B byteification evaluation suite

Adapted from the Base Easy Suite of Olmo 3^28^. Tasks were formatted as rank choice (RC, following the setup of Gu et al.^103^) or as greedy continuation (Greedy Cont.). ICL: number of in-context examples; Acc: accuracy; # sub: number of subtasks. ^*a*^few-shot examples are built into the task; ^*b*^selected core tasks.

**Extended Data Table 3:**
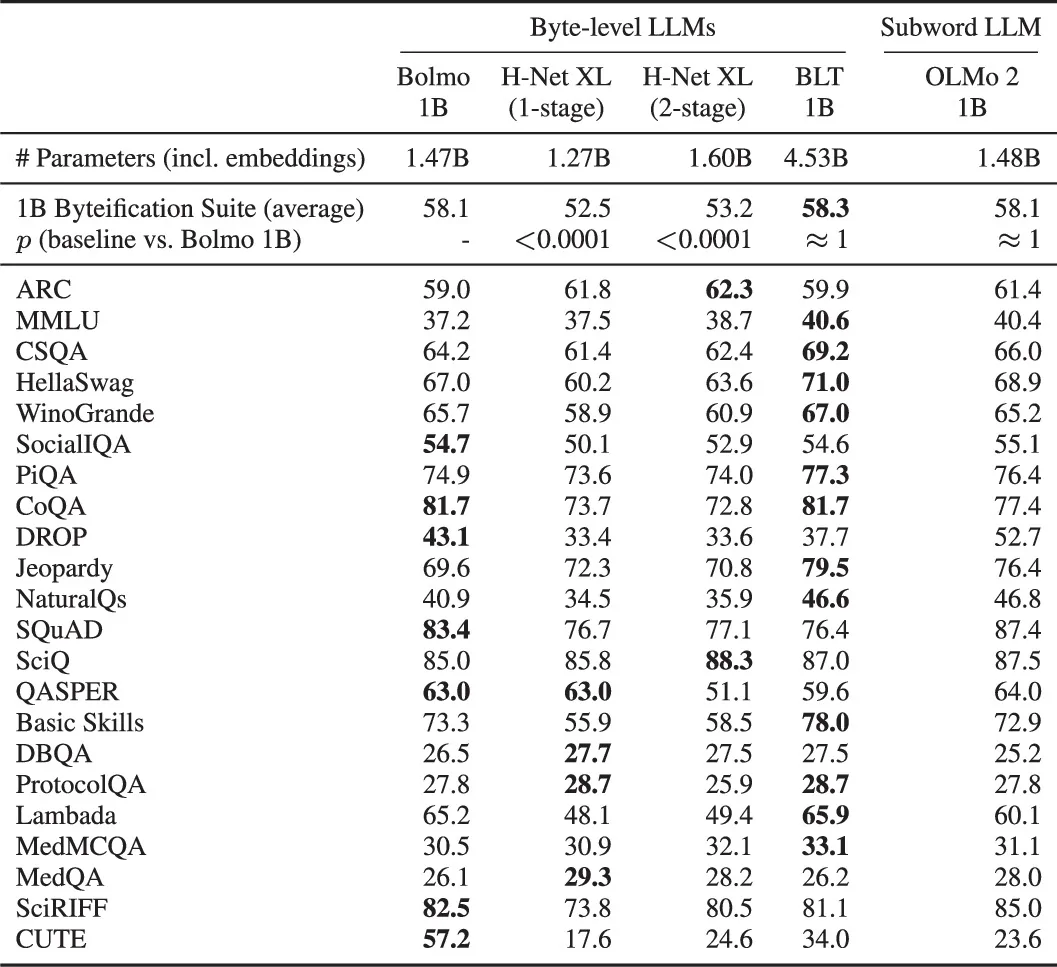
Bolmo 1B vs. existing byte-level LLMs of comparable size

All models except Bolmo were trained from scratch. Boldface indicates the best result per row among the byte-level LLMs; the subword-level LLM is shown for reference and is excluded from this comparison. *p*: Holm-Bonferroni corrected probability of the baseline meeting or exceeding Bolmo 1B over 10,000 bootstrap resamples. We include BLT 1B for reference although it contains substantially more than 1B parameters.

**Extended Data Table 4:**
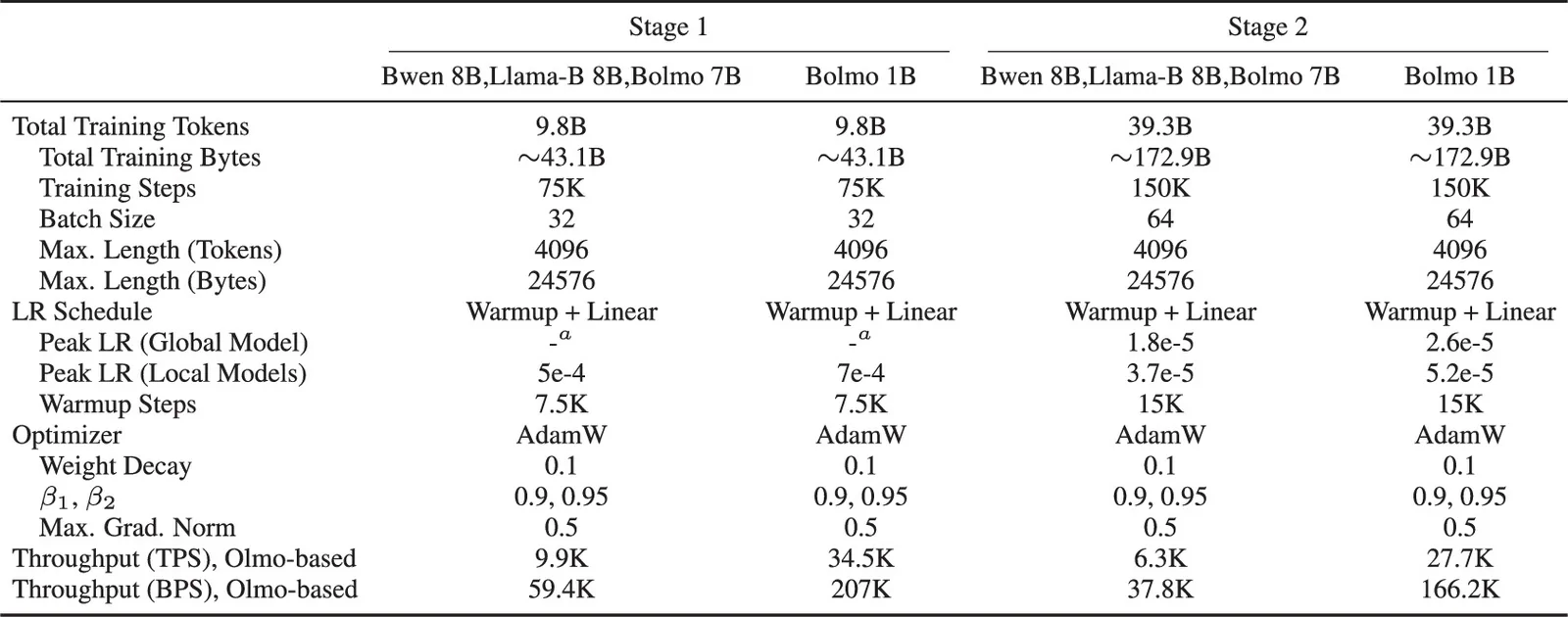
Training details

The two byteification stages are shown side by side. Rows are indented to indicate the hierarchy of settings. Throughput estimates are per accelerator, as achieved by the final Bolmo training runs on H100 GPUs. LR: learning rate; TPS: tokens per second; BPS: bytes per second. ^*a*^the global model is kept frozen in Stage 1, so no global-model learning rate applies; the Stage 1 peak learning rate governs the local encoder, local decoder, boundary predictor and LM head.

**Extended Data Table 5:**
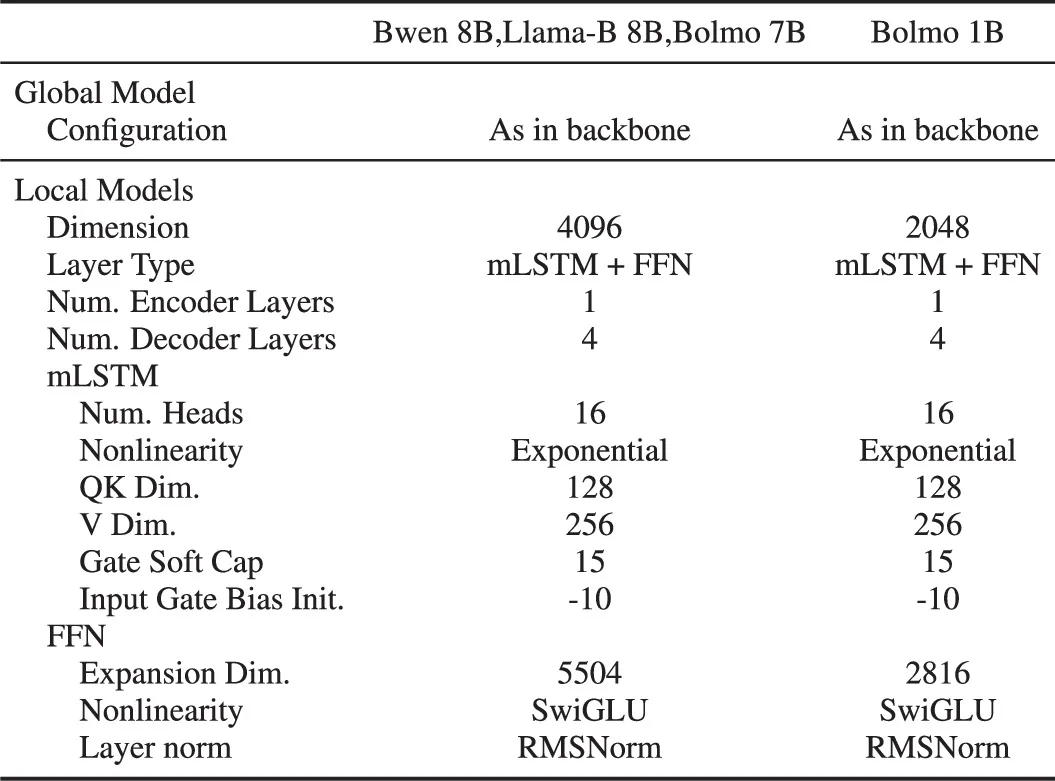
Architecture details

Rows are indented to indicate the hierarchy of settings within each model component. FFN: feed-forward network; QK: query-key; V: value.

**Extended Data Table 6:**
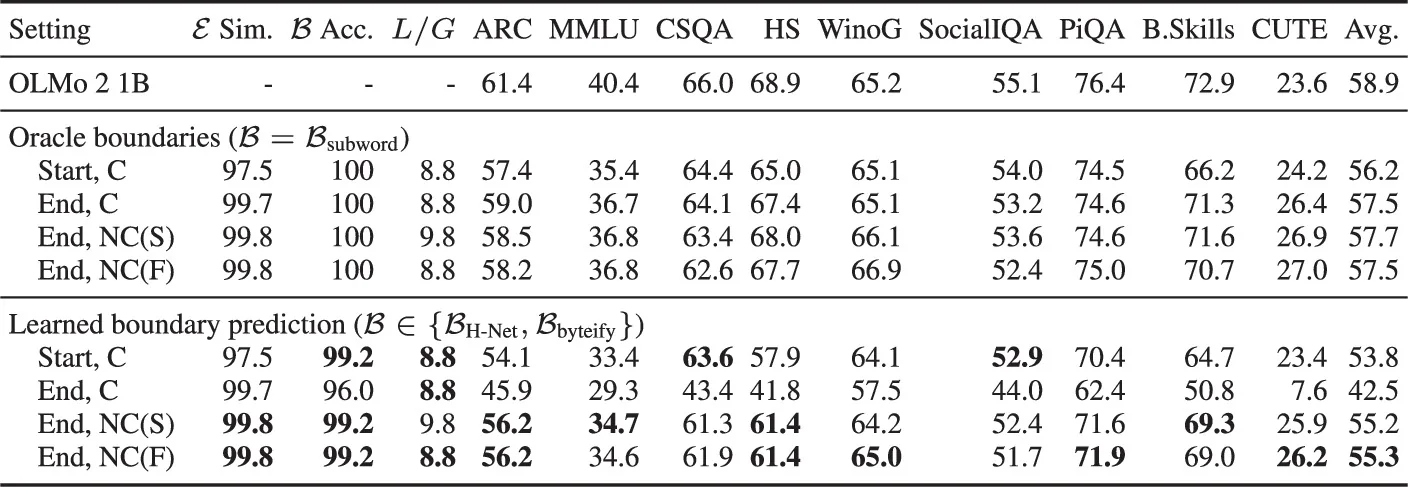
Comparison of boundary predictors after Stage 1 training

Models predict the patch start causally (Start, C), the patch end causally (End, C), the patch end non-causally using a separate boundary symbol (End, NC(S)), and the patch end non-causally with fused boundaries (End, NC(F)), our chosen setting. Causal boundary predictors can achieve either accurate boundaries or accurate representations; non-causal boundaries enable both. Boldface indicates the best result per column among the learned boundary prediction settings; the source subword-level LLM (OLMo 2 1B) and the oracle-boundary settings are shown for reference and are excluded from this comparison. ${\mathcal{E}}$ Sim.: cosine similarity of the pooled local encoder representations to the corresponding subword embeddings; ${\mathcal{B}}$ Acc.: accuracy of the boundary predictor; *L*/*G*: average number of local model invocations per global model invocation; HS: HellaSwag; WinoG: WinoGrande; B.Skills: Basic Skills; Avg.: average over the nine tasks.

## Data Availability

All datasets used in this study are publicly available. Training data are available at https://huggingface.co/datasets/allenai/bolmo_mix. All evaluation data are available at GitHub (https://github.com/allenai/olmes), with the exception of the character-understanding datasets CUTE and EXECUTE, which are available at https://huggingface.co/datasets/leukas/cute and https://huggingface.co/datasets/benjamin/execute, respectively.

## Code availability

The code needed to reproduce our results, including training code, is available at GitHub (https://github.com/allenai/bolmo-core). Our code is implemented in Python using PyTorch and based on the OLMo-core training framework, which is available at GitHub (https://github.com/allenai/olmo-core).
