# Supplementary material for: Retrofitting language models to operate over bytes
Source: Nature. 2026 Oct 7;658(8137):915–22. doi: 10.1038/s41586-026-11111-4 (PMC13686933; doi:10.1038/s41586-026-11111-4)
Supplement: Supplementary file 1 [file 41586_2026_11111_MOESM1_ESM.pdf]

nature portfolio

https://doi.org/10.1038/s41586-026-11111-4

Supplementary information

Retrofitting language models to operate over bytes

In the format provided by the authors and unedited

Supplementary Information for "Retrofitting Language Models to Operate Over Bytes"

Benjamin Minixhofer[1,2*], Tyler Murray[1], Tomasz Limisiewicz[3], Anna Korhonen[2], Luke Zettlemoyer[3], Noah A. Smith[1,3], Edoardo M. Ponti[4], Luca Soldaini[1], Valentin Hofmann[1,5,6]

[1]Allen Institute for AI, Seattle, WA, USA
[2]Language Technology Lab, University of Cambridge, Cambridge, UK
[3]University of Washington, Seattle, WA, USA
[4]Imperial College London, London, UK
[5]LMU Munich, Munich, Germany
[6]Munich Center for Machine Learning, Munich, Germany

Contents

Future Directions 2

Exactness of the Stage 1 Objective 3

References 5

[*]Corresponding author. E-mail: bm644@cam.ac.uk.

Future Directions

We believe our byteification procedure enables a number of future research directions, bits of which are sketched below.

Bit 0. Investigating how architectures optimized for byteification perform when training from random initialization. We have restricted ourselves purely to the byteification setting. For example, we have not assessed how non-causal patch boundaries perform when training from random initialization. We expect that the increased expressivity of the boundary predictor might be generally useful, but we do not yet know.

Bit 1. Learning non-causal boundaries end-to-end. We have purely trained our boundary predictor through direct external supervision — either to match subword tokens, or to match merges over subword tokens. We believe a highly promising area is learning non-causal boundary predictors end-to-end. For example, boundaries could be learned end-to-end during post-training of a byteified model via RL, or by adapting methods like H-Net's[1] method of enabling gradient flow through the boundary predictor to the non-causal setting.

Bit 2. Scaling patch size and local model capacity. We have designed the local models to minimize inference speed degradation when keeping the same patch size as the original subword model, since we have focused mainly on byteifying while keeping the patching constant. However, jointly using larger local models and a larger patch size might yield a better performance vs. efficiency tradeoff, as suggested by Pagnoni et al.[2] and Huang et al.[3].

Bit 3. Multi-byte prediction. While multi-token/byte prediction has been used to great effect to speed up language models[4–6], our models only predict the direct next byte. It is not clear how many sequential invocations of the global model multi-byte prediction could save; however, even saving sequential local model computations could lead to substantial speedups and permit larger local models, synergizing with Bit 2.

Bit 4. Non-destructive byteification. The remaining gap between the performance of our byteified models and the source subword models can to a large extent be attributed to the continued training setup generally hurting performance. Investigating ways to make continued training less destructive, such as PEFT methods[7,8], could be promising.

Bit 5. Specialized LTLM sampling methods. Subword-level language models have benefited from a range of sampling methods that have been to various extents designed for, or at least empirically validated on, predominantly subword-level LLMs[9–11]. We have not investigated how these methods transfer to LTLMs. Developing specialized sampling methods for LTLMs, for instance by adjusting the sampling strategy based on the position of the current byte within the patch, is also an intriguing topic.

Bit 6. Multilinguality and multimodality. Our models operate over UTF-8 bytes, a highly Latin-centric unit[12], and its training data mix is focused on English and coding data. Multilinguality and multimodality are natural next frontiers for Bolmo-like models. For example, more equitable input units[12,13] may lead to better and fairer multilingual LTLMs.

Bit 7. Batched inference optimizations. We have shown that our byteified models can achieve throughputs competitive with subword-level LLMs in the batchsize = 1 setting, which is sufficient for edge applications. However, achieving fast batched inference of LTLMs by applying, e.g., PagedAttention[14] and continuous batching[15] will be necessary to unlock a wider range of applications. Here, there are some additional challenges for LTLMs caused by their dynamicity (a fixed number of tokens across examples causes a variable number of bytes and vice versa), which require additional work (to our knowledge, the only investigation into efficient batched LTLM inference so far is through Aleph Alpha's vllm fork).

Exactness of the Stage 1 Objective

Here, we show that the Stage 1 distillation objective (see Methods, "Byteifying Procedure" in the main text) is *exact*: assuming a frozen global model $\mathcal{M}$ and sufficient capacity in the local encoder, local decoder, boundary predictor and LM head, if $\mathcal{L}_{\mathcal{B}}$, $\mathcal{L}_{\mathcal{E}}$ and $\mathcal{L}_{\mathcal{D},\text{Distill}}$ are all minimized on the data distribution $\mathcal{D}$, then the retrofitted byte-level model assigns the same likelihood as the original subword model to every text $x \in \text{supp}(\mathcal{D})$.

Preliminaries. To compare a byte-level and a subword-level model on the same data, we treat tokenization as *external* to the model and write each token as a sequence of bytes plus a boundary symbol, $t = \{x_0, x_1, \ldots, x_n, b\}$. The next-token probability is then the joint probability of the next bytes and those bytes being followed by a patch boundary; a subword-level LLM thus implicitly predicts the location of the next patch boundary. To compute the probability of the next bytes *without* the patch boundary, we would have to marginalize over all tokens with prefix $\{x_0, \ldots, x_n\}$ (see also Vieira et al.[16]), which is what generally makes likelihood comparisons across tokenizations difficult. Our byteified models sidestep this by predicting the patch boundary symbol explicitly, so the per-patch byte likelihood and the per-token subword likelihood model the same event.

Under this view, the subword model assigns to a text $x \in \text{supp}(\mathcal{D})$ the likelihood

$$P_{\text{subword}}(x) = \prod_i \text{LMHead}_{\text{subword}}(z_{\text{subword}})[i, \text{next\_tok}(x, i)],$$

with $z_{\text{subword}} = \mathcal{M}(\mathcal{T}_{\text{subword}}(x))$. The retrofitted model assigns

$$P_{\text{byte}}(x) = \prod_i \prod_{j \in T(x,i)} \text{LMHead}(\hat{z})[j, \text{next\_byte}(x, j)],$$

where the inference-time byte representations are

$$\hat{h} = \mathcal{M}(\text{Pool}(\mathcal{E}(\mathcal{T}(x)), \mathcal{B}_{\text{byteify}}(\hat{e}))), \qquad \hat{z} = \mathcal{D}(\text{Depool}(\hat{e}, \hat{h}, \mathcal{B}_{\text{byteify}}(\hat{e}))),$$

with $\hat{e} = \mathcal{E}(\mathcal{T}(x))$ the encoded byte representations and the third argument of Depool the boundary information specifying which patch representation to use at each byte position. Our goal is to show $P_{\text{byte}}(x) = P_{\text{subword}}(x)$ for every $x \in \text{supp}(\mathcal{D})$.

Step 1: Boundary Equivalence. Since $\mathcal{B}_{\text{subword}}(x)_t \in \{0, 1\}$, the binary cross-entropy $\mathcal{L}_{\mathcal{B}}$ is zero iff $\mathcal{B}_{\text{byteify}}(\hat{e})_t = \mathcal{B}_{\text{subword}}(x)_t$ for every t (treating $\mathcal{B}_{\text{byteify}}$'s outputs as hard $\{0, 1\}$ at the minimum). Hence

$$\mathcal{L}_{\mathcal{B}} = 0 \;\Rightarrow\; \mathcal{B}_{\text{byteify}}(\hat{e}) = \mathcal{B}_{\text{subword}}(x). \tag{1}$$

In particular, the bytes-plus-boundary data modeled by the retrofitted model coincides exactly with the data modeled by the original subword model.

Step 2: Hidden-State and LMHead Equivalence. Recall

$$Y_{\mathcal{E}} = \mathcal{M}_{:n}(\mathcal{T}_{\text{subword}}(x)),$$
$$\hat{Y}_{\mathcal{E}} = \mathcal{M}_{:n}(\text{Pool}(\mathcal{E}(\mathcal{T}(x)), \mathcal{B}_{\text{subword}}(x))),$$

and $\mathcal{L}_{\mathcal{E}} = \|Y_{\mathcal{E}} - \hat{Y}_{\mathcal{E}}\|$. Since the frozen subsequent layers $\mathcal{M}_{n+1:}$ are a deterministic function applied to both sides, $Y_{\mathcal{E}} = \hat{Y}_{\mathcal{E}}$ implies

$$\mathcal{M}(\text{Pool}(\mathcal{E}(\mathcal{T}(x)), \mathcal{B}_{\text{subword}}(x))) = \mathcal{M}(\mathcal{T}_{\text{subword}}(x)) = z_{\text{subword}}.$$

Using (1) to substitute $\mathcal{B}_{\text{subword}}(x)$ with $\mathcal{B}_{\text{byteify}}(\hat{e})$ on the left, we obtain the inference-time hidden state

$$\hat{h} = \mathcal{M}(\text{Pool}(\mathcal{E}(\mathcal{T}(x)), \mathcal{B}_{\text{byteify}}(\hat{e}))) = z_{\text{subword}}. \tag{2}$$

Applying (1) again to the boundary argument of Depool and composing with the deterministic Depool, $\mathcal{D}$ and LMHead yields

$$\text{LMHead}(\hat{z}) = \text{LMHead}(\hat{z}_{\text{subword}}), \tag{3}$$

where $\hat{z}_{\text{subword}} = \mathcal{D}(\text{Depool}(\hat{e}, z_{\text{subword}}, \mathcal{B}_{\text{subword}}(x)))$ is the training-time byte representation used by $\mathcal{L}_{\mathcal{D},\text{Distill}}$.

Step 3: Per-Patch Likelihood Equivalence. The temperature-modulated binary cross-entropy f has, for any target $y \in [0,1]$, a unique minimum in $\hat{y}$ at $\hat{y} = y$. Hence, at any x on which $\mathcal{L}_{\mathcal{D},\text{Distill}}$ is minimized,

$$\prod_{j \in T(x,i)} \text{LMHead}(\hat{z}_{\text{subword}})[j, \text{next\_byte}(x,j)] = \text{LMHead}_{\text{subword}}(z_{\text{subword}})[i, \text{next\_tok}(x,i)] \quad \forall i. \tag{4}$$

Note that (4) only constrains the *product* of byte probabilities over a patch; the per-byte conditionals inside a patch are not uniquely determined by the loss — *exactness* refers to the text-level likelihood, not to per-byte conditional distributions. Prior LTLMs without output boundary prediction would not enable exact distillation in this way since they cannot factor in the patch boundary likelihood.

Conclusion. Combining (3) with (4) gives, for every i,

$$\prod_{j \in T(x,i)} \text{LMHead}(\hat{z})[j, \text{next\_byte}(x,j)] = \text{LMHead}_{\text{subword}}(z_{\text{subword}})[i, \text{next\_tok}(x,i)].$$

Taking the product over i,

$$\begin{aligned} P_{\text{byte}}(x) = &\prod_i \prod_{j \in T(x,i)} \text{LMHead}(\hat{z})[j, \text{next\_byte}(x,j)] = \\ &\prod_i \text{LMHead}_{\text{subword}}(z_{\text{subword}})[i, \text{next\_tok}(x,i)] = P_{\text{subword}}(x), \end{aligned} \tag{5}$$

for every $x \in \text{supp}(\mathcal{D})$, as desired. We have omitted $\mathcal{L}_{\mathcal{D},\text{CE}}$ throughout since adding it forgoes exactness (see Methods, "Byteifying Procedure" in the main text).

References

1. Hwang, S., Wang, B. & Gu, A. Dynamic chunking for end-to-end hierarchical sequence modeling. In *Proc. Fourteenth International Conference on Learning Representations* (2026).

2. Pagnoni, A. et al. Byte latent Transformer: Patches scale better than tokens. In *Proc. 63rd Annual Meeting of the Association for Computational Linguistics (Volume 1: Long Papers)*, 9238–9258 (Association for Computational Linguistics, Vienna, Austria, 2025).

3. Huang, H. et al. Over-tokenized Transformer: Vocabulary is generally worth scaling. In *Proc. 42nd International Conference on Machine Learning*, vol. 267 of *Proceedings of Machine Learning Research*, 26261–26282 (PMLR, 2025).

4. Gloeckle, F., Idrissi, B. Y., Roziere, B., Lopez-Paz, D. & Synnaeve, G. Better & faster large language models via multi-token prediction. In *Proc. Forty-first International Conference on Machine Learning* (2024).

5. Cai, T. et al. Medusa: Simple LLM inference acceleration framework with multiple decoding heads. In *Proc. Forty-first International Conference on Machine Learning* (2024).

6. Grivas, A. et al. Fast and expressive multi-token prediction with probabilistic circuits. Preprint at https://arxiv.org/abs/2511.11346 (2025).

7. Hu, E. J. et al. LoRA: Low-rank adaptation of large language models. In *Proc. International Conference on Learning Representations* (2022).

8. Pfeiffer, J., Ruder, S., Vulić, I. & Ponti, E. Modular deep learning. *Transactions on Machine Learning Research* (2023).

9. Holtzman, A., Buys, J., Du, L., Forbes, M. & Choi, Y. The curious case of neural text degeneration. In *Proc. International Conference on Learning Representations* (2020).

10. Meister, C., Pimentel, T., Wiher, G. & Cotterell, R. Locally typical sampling. *Transactions of the Association for Computational Linguistics* **11**, 102–121 (2023).

11. Minh, N. N. et al. Turning up the heat: Min-p sampling for creative and coherent LLM outputs. In *Proc. Thirteenth International Conference on Learning Representations* (2025).

12. Limisiewicz, T., Blevins, T., Gonen, H., Ahia, O. & Zettlemoyer, L. MYTE: Morphology-driven byte encoding for better and fairer multilingual language modeling. In *Proc. 62nd Annual Meeting of the Association for Computational Linguistics (Volume 1: Long Papers)*, 15059–15076 (Association for Computational Linguistics, Bangkok, Thailand, 2024).

13. Land, S. & Arnett, C. BPE stays on SCRIPT: Structured encoding for robust multilingual pretokenization. Preprint at https://arxiv.org/abs/2505.24689 (2025).

14. Kwon, W. et al. Efficient memory management for large language model serving with PagedAttention. In *Proc. 29th Symposium on Operating Systems Principles* (Association for Computing Machinery, 2023).

15. Yu, G.-I., Jeong, J. S., Kim, G.-W., Kim, S. & Chun, B.-G. Orca: A distributed serving system for Transformer-based generative models. In *Proc. 16th USENIX Symposium on Operating Systems Design and Implementation (OSDI 22)*, 521–538 (USENIX Association, Carlsbad, CA, 2022).

16. Vieira, T. et al. From language models over tokens to language models over characters. In *Proc. Forty-second International Conference on Machine Learning* (2025).
