# Supplementary material for: Retrofitting language models to operate over bytes
Source: Nature. 2026 Oct 7;658(8137):915–22. doi: 10.1038/s41586-026-11111-4 (PMC13686933; doi:10.1038/s41586-026-11111-4)
Supplement: Supplementary file 2 [file 41586_2026_11111_MOESM2_ESM.pdf]

nature portfolio

Peer Review File

Retrofitting Language Models to Operate Over Bytes

Corresponding Author: Mr Benjamin Minixhofer

Any redactions in this file are there to maintain patient confidentiality, the confidentiality of unpublished data, or to remove third-party material.

This file contains all reviewer reports in order by version, followed by all author rebuttals in order by version.

Version 0:

Reviewer comments:

Referee #1

(Remarks to the Author)
Summary of the key results

Current LLMs rely on subword tokenization, which limits character-level understanding. Byte-level LLMs were proposed to close this gap, but their performances lag significantly behind subword models. This paper proposes "byteification," converting a pretrained subword LLM into a byte-level model rather than training from scratch. The method involves: (1) A new component for the model architecture, non-causal boundary predictor, that uses one byte of future context; (2) a two-stage training procedure, first distilling the source model's behavior into new local components, then end-to-end fine-tuning, using approximately 39.3B tokens in total. The resulting models are Bolmo 7B (from OLMo 3 7B) and Bolmo 1B (from OLMo 2 1B).

Bolmo 7B reports higher average scores than prior byte-level LLMs (EvaByte 6.5B, TFree-Hat 7B, BLT 7B), while remaining below the source OLMo 3 7B on most standard benchmarks. The largest gains are on character understanding tasks (CUTE, EXECUTE). The authors also explore higher compression ratios for faster inference and use Task Arithmetic to transfer post-training capabilities from subword checkpoints.

Originality

The main novelty of this paper is the byteification paradigm, which allows to quickly distill a pretrained subword LLM into a byte-level model. Besides a training procedure, the model architecture is also modified for this purpose. Other than the above, the rest of the approach is mainly inherited from exiting studies, including the LTLM architecture.

Significance

This paper is a significant step of byte-level LLM research, as it substantially lowers the cost of training byte-level LLMs and this is the first time that the performance of byte-level LLMs approaches that of subword models.

However, this advance is yet not a turning point for the byte-level models to replace the subword models, at least based on the experimental results in this paper. The experiments concerns mainly two aspects: effectiveness and efficiency. For the former, the byte-level model Bolmo still underperforms the subword model Olmo 3 on most benchmarks, and the only two benchmarks where they outperform are character understanding tasks. Note that though the paper reports Bolmo performs slightly better than the subword model Olmo 3 on code tasks, this improvement is actually an average pass@1 and pass@16, and on the practically more useful pass@1, Olmo 3 still outperforms Bolmo. Considering that the importance of character understanding cannot match the importance of the other tasks measured in the evaluation, the practical usefulness of byte-level models is limited.

For the efficiency aspect, the paper claims that byte-level models are more flexible in improving the compression ratio for faster inference. However, the efficiency is measured in inference time with batch_size = 1. This could be reasonable for edge-side inference, but in most real-world applications, the inference occurs in cloud servers, and such a metrics cannot reflect the real efficiency of the models. I can imagine many challenges when optimizing Bolmo for batching. For example,

the dynamically changing difference between token length and byte length could lead to idle waiting, and three components (mLSTM->Transformer->mLSTM) increase memory access cost. This limitation is also discussed in Bit 7 of future work. Further considering that Bolmo is still slightly slower than Olmo 3 when at the same compression ratio level, as a result, at least the current result has not demonstrated the superiority of byte-level models in efficiency.

Data, Methodology, and Results

This paper presents a comprehensive set of experiments to demonstrate different aspects of the proposed approach. The use of statistics and treatment of uncertainties is appropriate as far as I can tell. However, when describing the results, the way the results are presented is significantly biased from an academic point of view.

1. The primary results table (Table 1) compares Bolmo 7B against the original Olmo 3 7B, which has not undergone continued training on the Bolmo data mix. The fairer baseline, Olmo 3 CT (continued training on the same data for the same number of gradient updates), is relegated to Appendix A (Table 4). This matters because Table 4 reveals that the suite-level gap between Bolmo 7B and Olmo 3 CT is negligible (62.4 vs. 61.9), and on most standard benchmarks Olmo 3 CT actually outperforms Bolmo (e.g., Math 49.8 vs. 48.9, MC Non-STEM 76.6 vs. 75.8).

2. The paper's headline result, character understanding, is similarly confounded: the large CUTE improvement (78.6 for Bolmo vs. 56.9 for Olmo 3) is substantially driven by 75M tokens of synthetic CUTE-style training data (Appendix C), and the authors acknowledge that "byte-level models otherwise do not acquire this knowledge through our short training schedule." Olmo 3 CT trained on the same data already reaches 72.9 on CUTE, leaving a marginal byteification gain of only 5.7 points. EXECUTE shrinks from an apparent +16.5 points (vs. Olmo 3) to +2.4 points (vs. Olmo 3 CT). The continued-training baseline should appear in the main table, and a controlled ablation removing the synthetic character data from the Bolmo training mix would be needed to isolate the true architectural contribution.

3. Similarly, when summarizing the overall performance in the abstract and in the introduction, the paper selects data where Bolmo performs better, and ignore the cases where Bolmo performs worse. For example, as mentioned before, Bolmo performs significantly worse than Olmo 3 on the important metrics pass@1 for most code tasks, yet the introduction only states the improvement on average but ignores the degradation on pass@1. The degradation on all other tasks except for character understanding is also ignored.

Furthermore, it is also desirable to further evaluate the generality of Byteification. Byteification is demonstrated exclusively on OLMo 2 and OLMo 3. It remains unclear whether the approach transfers to architecturally different model families (e.g., Llama, Mistral, Qwen), which may differ in embedding structure, attention patterns, or training dynamics. The embedding rank analysis in Appendix E hints at potential difficulties (the authors note that different models have different embedding rank structures), but no cross-family experiments are conducted. Given that the paper positions byteification as a general paradigm, this is a notable gap.

Clarity and Context

While the paper is overall understandable and easy to follow, the presentation of the introduction could be significantly improved.

The introduction focuses on the shortcoming of the subword models, and lists four main problems of the subword models. This gives the reader an impression that this paper is to propose a novel approach that is a significant advance over subword models. However, as discussed above, the evidences in this paper do not support this conclusion. In fact, regarding the four shortcoming, only the first one (losing character information) is demonstrated in the evaluation (with around 2.4%-5% advantage, but disadvantages in other domains). The last one (rigid computation allocation) is evaluated, but as discussed before, the evaluated scenario does not reflect the main application scenario. The remaining two (tokenization bias and English-centricity) are not evaluated at all. Furthermore, neither losing character information nor rigid computation allocation is a problem resolved in this paper, but is already resolved in existing approaches. The main contribution of this paper is resolve the problems in existing byte-level models. Therefore, in my opinion, the introduction should focus on the shortcomings of existing byte-level models.

References

The references include most related work. The authors may consider including studies in software engineering domains that show that losing character information is a problem in understanding abbrevation in variable names and propose character-level (similar to byte-level) models to address it.

Minor Issues

- Table 2: "p (vs. Bolmo 7B)" should read "p (vs. Bolmo 1B)" (copy-paste error from Table 1).
- Footnote 2: refers to "problem (ii)" but describes inefficiency in non-English languages, which is problem (iii); problem (ii) is tokenization bias.
- Appendix A uses "bytefying" twice (in the boundary predictor analysis and continued-training paragraphs), inconsistent

with "byteifying" used elsewhere.
- Section 3.1, Depooling paragraph: "providing a contextualized representations" — article/number disagreement.
- Section 4: the "Data" subheading is missing a period, unlike all other subheadings in the same section ("Model.", "Training.", "Baseline.", etc.).

(Remarks on code availability)

Referee #2

(Remarks to the Author)
I co-reviewed this manuscript with one of the reviewers who provided the listed reports.

(Remarks on code availability)

Referee #3

(Remarks to the Author)
Summary:
This paper proposes Bolmo, a family of byte-level LLMs based on Olmo model series. The authors propose to adapt a pretrained subword-baed Olmo model to byte-based model using a two-stage distillation process. Stage 1 mainly focuses on making sure the new initialized parameters for boundary predictor and byte-level patch aggregators match the base subword model. Stage 2 jointly tunes all parameters on language-modeling tasks to enable flexible byte-level boundaries. The authors evaluate this approach at 1B and 7B parameter scales, which shows that Bolo models are able to achieve significant improvements on character-level reasoning benchmarks while remain comparable on other tasks as the base model.

Originality:
This paper has some novel ideas regarding adapting a pretrained subword-based model to character-level model. The main novelties are: 1. Non-causal boundary prediction at prefill time to address the capacity difference of subword and character-based LLMs; 2. A two-stage distillation framework that recovers the performance of subword-based pretrained models with much less compute than training from scratch.
However, the idea of byte-level LLMs are not new, and this paper built upon the prior work on byte-level and character-level models.
The paper outlines a computationally efficient approach for converting subword-based models to character-level models. However, the work only shows improved performance on a limited set of character-level tasks that are somewhat synthetic. The lack of practical performance gains might limit the significance of the work.

Data and method:
The approach is clearly document and valid. The non-causal byte-level boundary prediction is well-motivated and reasonable, especially at prefill time.
The training data consists of general pretraining corpus and some CUTE-style synthetic data for character understanding. However, the use of CUTE-style pretraining data introduces some uncertainty about the gains on character-level reasoning tasks. It's not clear whether the improvement comes from character-level modeling, or the adaptation over these pretraining data.

Appropriate use of statistics:
The authors use bootstrap resampling (10k samples) and Holm–Bonferroni correction when comparing Bolmo to baselines.

Conclusion:
The paper shows experimental results supporting that the proposed method allows efficient adaptation of subword-based pretrained models to byte-level LLMs. The adapted models are shown to match the performance of the subword-based models on most tasks, while performing better on character-level reasoning tasks and coding tasks. However, the adaptation data contains some synthetic data similar to the character-level reasoning tasks, which makes comparison to the baseline subword-based models seems a bit unfair.

Improvements:
1. It might be more thorough to continue pretrain the baseline subword-based models on the data mixture, so that you could show the gains of the byte-level model comes from the change in model architecture instead of additional training
2. The performance gains of the byte-level models are still a bit limited in terms of tasks and domains. Would it be possible to show some improvements in more practical use cases? For example, on multilingual or multimodal tasks.

(Remarks on code availability)
The implementation seems very thoroughly documented.

Referee #4

(Remarks to the Author)

A. Summary of the key results

A1.

The paper shows that a byteified model, Bolmo 7B, can substantially outperform prior public byte-level LLMs while avoiding large performance degradation relative to its source subword model, OLMo.

I use this wording because the paper makes stronger claims, such as "the first family of fully open byte-level LLMs achieving performance on the level of state-of-the-art subword-level LLMs" [L62-L63] and "the first fully open family of byte-level LLMs on par with or surpassing state-of-the-art subword-level LLMs at the 1B and 7B parameter scales" [L560-L562], but the main results section is more cautious: Bolmo 7B "statistically significantly outperforms all the prior byte-level models" [L382-L383] and "comes close to matching the performance of the source Olmo 3 model" [L384-L385]. I therefore limit the key result to superiority over prior public byte-level models and near-parity with the source subword model, rather than broader parity or superiority over subword LLMs in general.
Table 1 also reports equal suite scores for Bolmo 7B and Olmo 3 7B, with a p-value of "\approx 0.53". Moreover, Bolmo's gains appear to come mainly from the "Char" sub-category, while its performance on other sub-categories, such as Math, MC, and GenQA, which seems to be more common tasks, is worse than that of Olmo 3.

A2.

The paper shows that Bolmo improves character understanding and reports average improvement on coding tasks relative to the source Olmo 3 model.

This wording is close to the paper's own wording: Bolmo 7B "greatly outperforms the source Olmo 3 on character understanding and improves on average across a set of coding tasks" [L83-L84]. I still avoid a stronger formulation for two reasons. First, the training mix is explicitly "augmented with 75M tokens of CUTE-style data ... to encourage character understanding" [L352-L353], so this makes it difficult to attribute the result to byteification alone.
Second, for coding, the paper itself notes that the gain comes from "higher pass@16 rates at generally slightly lower pass@1" [L388] and adds that "we cannot conclude that byte-level models are fundamentally better suited to generating more diverse continuations" [L391-L392]. For that reason, I keep the claim at strong character gains and average coding improvement, without extending it to stronger causal or mechanistic interpretations.

A3.

The paper offers some useful empirical takeaways about byteification. For example, non-causal boundary prediction can improve downstream performance, Stage 1 helps but is not strictly necessary, and higher compression can improve inference efficiency.

This is a compressed summary of the paper's takeaways of Sections 6.1, 6.2, and 6.3. I intentionally soften stronger phrases such as "can be arbitrarily further sped up" [L84] and "which is only possible to a limited extent in subword-level LLMs" [L85] because the safest summary of the paper's key results should stay close to what is directly demonstrated rather than the paper's broadest prose claims.

B. Originality and significance: if not novel, please include reference

If my understanding is correct, this paper itself already acknowledges that some core components in Section 3 overlap with prior work, and therefore those parts may be better viewed as adapted ingredients rather than the main novelty of this paper.
In particular, the authors explicitly state that byteification itself is not new: "Byteification was first done by Pagnoni et al. (2025) ..." and "Hwang et al. (2025) later byteified by supervising the boundary prediction to match the subword boundaries" together with "introducing an auxiliary embedding-matching loss" [L149-L152].
The same pattern appears in some architectural details. For example, the paper says that pooling is "This is equivalent to the pooling done by Hwang et al. (2025)" [L184-L185] and that the depooling design is "This is similar to Hwang et al. (2025)'s depooling" [L193].
Likewise, for the non-causal boundary predictor, the authors note that "An equivalent parametrization, although using the current byte and one byte before, is used by Hwang et al. (2025)." [L227-L228].
Even the output-side boundary prediction idea is not presented as entirely unprecedented, since the paper notes: "It is worth noting that predicting the boundary symbol **is analogous to the output boundary prediction in Fleshman and Durme (2023), although the motivation differs." [footnote 11].**

Given these statements, my understanding is that the novelty of the methodological perspective should not be

framed as each individual component was newly proposed here in isolation.
Rather, the paper's contribution seems to lie in combining and adapting these ingredients into a system specifically suited to byteification. This interpretation is also consistent with the authors' own wording: "Our key contribution is creating an LTLM which is specifically suited to byteifying." [L152-L153].
For that reason, I would encourage the authors to revise the presentation so that it more clearly separates reused or closely related design elements from the actually novel aspects of the work. As written, a reader could easily come away with the impression that several Section 3 components are individually novel, even though the paper itself appears to acknowledge that an important part of the contribution lies in the particular reconstruction and integration of existing ideas for the byteification setting.

C. Data & methodology: validity of approach, quality of data, quality of presentation

I do not see any fundamental validity issue with the overall approach, although some technical details still need to be clarified.
Moreover, the evaluation is extensive enough to support the paper's main comparative claims against prior public byte-level baselines.
That said, some broader claims about generality and practical applicability are less fully established. For example, higher compression is shown mainly for 1B models, post-training transfer is demonstrated in only one case study, and speed is evaluated only at batch size 1 on an H100.

The main byteification experiments are conducted within the OLMo family (OLMo 3/OLMo 2), so it remains unclear how well the proposed method generalizes to other model families.
The paper would be strengthened if the authors could show that a similar tendency also holds for model families beyond OLMo.

Several important points in the paper still need further clarification (see the comments below).
Accordingly, the presentation could be improved to avoid misunderstandings and help readers develop a clear understanding of the proposed method, as the method is fairly complex and some parts are not easy to follow.

D. Appropriate use of statistics and treatment of uncertainties

This paper reports p-values in Tables 1 and 2, which summarize the central benchmark comparisons in the main results section.
According to the captions of Tables 1 and 2, these p-values are the Holm-Bonferroni corrected probabilities that baseline performance meets or exceeds Bolmo 7B (Table 1) or Bolmo 1B (Table 2) over 10k bootstrap resamples.
(That said, note that in Table 2, the notation "Bolmo 7B" should be "Bolmo 1B" because of a simple typo.)

Although the exact computational procedure is not fully specified in this paper (and I think the paper should present clear equations for the statistical significance tests used), the reported statistical testing appears reasonable at a high level and supports meaningful gains over some baseline systems.

E. Conclusions: robustness, validity, reliability

In conclusion, the authors state, "Overall, byteifying finally makes byte-level LLMs a practical choice competitive with subword-level LLMs, and enables future research directions on byte-level LLMs for both the byteification setting and training from scratch." [L567-L569]

However, this claim appears to go somewhat beyond what the paper's experimental results directly support.
For example, Table 1 shows that Bolmo 7B achieves overall performance roughly on par with Olmo 3.
At the same time, the largest positive difference relative to Olmo 3 appears in the "Char" sub-category, with a smaller positive difference in "Code", while Bolmo 7B underperforms Olmo 3 in several other categories, including GenQA, Math, and both MC sub-categories.

This suggests that the overall parity with Olmo 3 may be driven largely by the substantial gains in the Char sub-category, even though its performance remains lower in several more conventional benchmark categories.
Given this, the broader conclusion that byte-level LLMs can compete effectively with subword-level LLMs appears somewhat stronger than the comparisons with Olmo 3 directly warrant.

I do think this paper makes several valuable points.
However, for it to qualify as a top-tier scientific contribution, I think several aspects remain unconvincing, since some of the claims are stronger than the experimental results directly support.

F. Suggested improvements: experiments, data for possible revision

F1.
The definition of the local encoder loss L_E appears to contain a parenthesis error: $M_{:n}(\cdot)$ and the norm in $\mathcal{L}_{\mathcal{E}} := \| \mathcal{M}_{:n} (\mathrm{Pool}(\mathcal{E}(\hat{e}, B_{\mathrm{subword}}(x))) - \mathcal{M}_{:n}(T_{\mathrm{subword}}(x)) \|$. I recommend correcting the parentheses and explicitly rewriting it as a norm of the difference between two quantities. [L298-L299]

F2.
Comment on Stage 2 loss notation: The notation for the Stage 2 objective is inconsistent. The text first refers to the cross-entropy term as $L_{D,CE}$, then says it will be referred to as L_{CE}, and finally defines $L_{Stage2} \coloneqq \lambda_B L_B + \lambda_{CE} L_{CE}$. I suggest standardizing this notation throughout to avoid confusion. [L341-L345]

F3.
The claim that the decoder distillation objective $L_{D,Distill}$ is “exact” is not fully convincing from the current presentation. The loss compares patch probabilities using the comparison function $f(\cdot \parallel \cdot)$ with temperature-scaled terms, but it is not obvious from the equations alone why this yields the stated if-and-only-if exactness guarantee.
Please provide either a short proof, a clearer derivation, or a more qualified statement.

F4.
The role of the comparison function $f(\hat{y} \parallel y)$ is not sufficiently clear.
In particular, the equation uses a binary-cross-entropy-like form between two probabilities, but it is hard to tell which argument is intended to be the prediction and which is the target, especially since the surrounding text does not define the argument order carefully. Please clarify the semantics of f and the meaning of each argument.

F5.
Regarding SuperBPE, the authors pointed out that "we can speed up the subword-level LLM while retaining performance to a large extent. However, at some vocabulary size threshold, the subword-level LLM breaks off the frontier as the softmax begins to dominate the FLOPs (for OLMo 2 1B, this is somewhere between a vocabulary size of 200k and 400k tokens)." [L448-L451]

This trend is also evident in Figure 3.
However, the discussion does not consider whether alternative output-layer techniques for reducing softmax cost might affect the frontier comparison.
In particular, a range of methods, such as hierarchical softmax, has long been available.
It would therefore be helpful for the authors to clarify whether their frontier comparison depends on the specific softmax implementation used in the subword baseline, and whether more efficient output-layer implementations might narrow the reported gap.

F6.
I am not sure I fully understand how decoding behaves when the byte-level output of the local decoder/LM head diverges from the continuation implicitly suggested by the current global patch representation, $\hat{h}$.

Unless I am missing something, the paper does not seem to explain what happens in this case.

My understanding of Section 3 is that, during decoding, the global model produces a continuous patch-level representation $\hat{h}$, which is then depooled into byte-level representations. The local decoder/LM head then predicts, at each step, both the next byte and whether a boundary follows. To make the question concrete, suppose the current global representation $\hat{h}$ corresponds to a patch continuation that is semantically closest to something like "flowerbed". After depooling, the model might generate "f", then "l", then "o", but then predict "a" rather than "w", yielding floa... instead of flow.... In that case, does decoding simply continue from the generated byte sequence, even though the current $\hat{h}$ may have been shaped by a different patch-level hypothesis? If so, is this kind of mismatch between the current global patch representation and the realized byte-level continuation explicitly allowed by the model design, or is there some mechanism intended to keep the two aligned?

More precisely, depooling computes $z = \mathrm{Depool}(\hat{e}, \hat{h}, p)$, but at that point there would seem to be a mismatch between $\hat{e}$ and $\hat{h}$, with $\hat{e}$ derived from "floa" and $\hat{h}$ from "flowerbed".

A related question concerns boundary prediction in this setting. If the model generates floa and then predicts a boundary immediately after "a," should this be understood as effectively ending the current patch at that point, even if the current global representation had implicitly favored a longer continuation such as "flowerbed"? In other

words, once a boundary is emitted, is the previous patch-level continuation simply discarded, with the next global patch representation computed from the newly completed patch? I could not find an explicit discussion of this decoding behavior in the current text, so I would appreciate clarification, perhaps with a short worked example.

More generally, this kind of mismatch between $\hat{h}$ and the LM head's prediction, including boundary prediction, may warrant further discussion. It would also be helpful to report how frequently such cases occur, together with concrete examples, to better illustrate the internal behavior of the proposed model, Bolmo.

F7.
I found the definition of $T_{\mathrm{SubwordSuffix}}(x_{:i})$ somewhat hard to parse, and I think a short, concrete example would help clarify its intended behavior.

My understanding is as follows: for each byte position i, one considers the prefix $x_{:i}$, examines its suffixes, and selects the longest subword from the original subword vocabulary that matches a suffix ending exactly at position i. The embedding of that subword is then added to the byte embedding at position i.

For example, suppose the current text prefix ends with "flower", and the current byte position i is the final "r". If the subword vocabulary contains candidates such as "r", "er", "wer", "ower", and "flower", would $T_{\mathrm{SubwordSuffix}}(x_{:i})$ select the embedding of "flower", since it is the longest subword matching the suffix ending at that byte? Likewise, if the current position were the "w" in "flow", would the method select the embedding of "flow" if that token exists in the vocabulary? If so, a short worked example in the paper might be helpful, since the current wording is somewhat difficult to parse at first reading.

Additionally, is my understanding correct that the model does not care whether the current word will ultimately be "flower" and instead uses the embedding of "flow" at the position of "w"?

F8.
A point that may merit clarification is how Bolmo's parameter count should be understood relative to that of the source OLMo model.

My reading is that Bolmo retains the source model's transformer backbone while replacing the original subword LM head with a new byte-level or fused-symbol output head.

If that interpretation is correct, then the original OLMo LM head parameters are presumably removed, so Bolmo's total parameter count would equal the source OLMo parameter count plus the newly introduced Bolmo-specific modules minus the original LM head parameters. I could not find an explicit discussion of this accounting in the paper. It would therefore be helpful to clarify whether this is the intended interpretation and, if so, to state explicitly how Bolmo's total parameter count relates to that of the source OLMo model.

F9.
Table 5 indicates that a substantial fraction of the reported Code / Math / GenQA results are based on sampling, often with "temp=0.6, top-p=0.6". The main text further states that the coding results are obtained under "sampling settings ... (temperature = 0.6, top_p=0.6; see Appendix B)" [L388-L393].
However, the paper does not clearly explain how top-p sampling is implemented in Bolmo's byte-level decoding. Specifically, the model predicts "the current byte and whether it is followed by a boundary at every step" using a fused vocabulary of "512 symbols" [L253-L257], but the paper does not state whether top-p is applied to this fused byte-level output at each decoding step, and it is not clear whether the throughput measurements in Figure 7 are based on greedy decoding or sampling [L539-L546].
Because this distinction could materially affect both efficiency and the interpretation of the sampling-based results, I encourage the authors to clarify the exact sampling procedure used for Bolmo and to specify the decoding policy used in the efficiency comparisons.

G. References: appropriate credit to previous work?

The following papers should also potentially be cited when discussing how the motivation, concepts, or methodologies in this paper differ from those of earlier studies.

[Altıntaş+, submitted to ICLR-2026] TokSuite: Measuring the Impact of Tokenizer Choice on Language Model Behavior
This paper introduces a suite of benchmarks and models designed to isolate the effects of tokenization by varying only the tokenizer while holding all other conditions constant, and reports the corresponding evaluation results. As prior work, it could support a more systematic and directly comparable assessment of Bolmo's tokenizer-related claims and may help qualify broader generalization claims.

[Xu+, ACL-2025] Enhancing Character-Level Understanding in LLMs through Token Internal Structure Learning
Motivated by the observation that BPE obscures the internal character structure of tokens, this paper proposes Token Internal Position Awareness (TIPA) to improve character-level understanding in subword LLMs.
This qualifies a strong reading of the present paper's character-understanding results by showing that similar gains may also be pursued in subword models.

[Dolga+, EMNLP-2025 Findings] From Characters to Tokens: Dynamic Grouping with Hierarchical BPE
To combine the strengths of character-level and subword tokenization, this paper proposes dynamic character grouping based on BPE structure as an alternative to existing dynamic patching strategies.
It suggests that the broader direction of dynamic grouping and patch-granularity control had already been explored outside the specific Bolmo formulation.

[Zhu+, ACL-2025 Findings] Unsupervised Morphological Tree Tokenizer
This paper proposes a tokenizer based on character-level structure induction.
It addresses a problem setting close to Bolmo's, namely, the possibility that subword tokenization can hide fine-grained information, and shows that a solution need not be byte-level.

[Hiraoka+, EMNLP-2025 Findings] Spelling-out is not Straightforward: LLMs' Capability of Tokenization from Token to Characters
This paper shows that, even in subword LLMs, although the embedding layer alone is insufficient, character knowledge is reconstructed in later intermediate layers.
As prior work, it adds evidence that character information can be reconstructed in subword LLMs, which qualifies a strong reading of the paper's claim that subword tokenization loses character information.

# H. Clarity and context: lucidity of abstract/summary, appropriateness of abstract, introduction and conclusions

The abstract, introduction, and conclusion are generally clear and appropriate, but a few statements appear somewhat stronger than what the experiments directly establish, as discussed in the comments above.

(Remarks on code availability)
I have tried to run the code and replicate some of the experiments.
Unfortunately, I did not have enough computational resources or time to replicate them.

At the very least, I can say that the code preparation is excellent.
This code repository would be very valuable for reproducing the experiments in future studies.

Version 1:

Reviewer comments:

Referee #1

(Remarks to the Author)
# Review (Second Round / Resubmission): Byteifying the Next Generation of Language Models

I thank the authors for a substantial revision, and several of my earlier concerns are now addressed: code is split into pass@1 and pass@16, the strongest performance claims have been toned down, the introduction is better balanced, and the recipe is validated on a second family (Qwen 3 8B → Bwen 8B). Yet some concerns remain, and an inconsistency between the two versions is not explained. I go through these below in the order of my original review.

## Summary of the key results

A newly added result in this revision is the Bwen 8B model, obtained by byteifying Qwen 3 8B Base, showing the generality of byteification.

## Originality

The core novelty of this paper, the byteification paradigm for supporting distilling, remains unchanged. Yet this version does a better job to present the contribution.

Significance

Compared to the previous version, the signficance of this paper slightly improved as the new experiments show that the approach generalizes beyond the OLMo family. Yet the main limitations of this paper remains: the paper still does not show is that byte-level models can replace subword ones, on either effectiveness or efficiency.

Effectiveness: the paper shows that byte-level models and subword models are complementary, but byte-level models are not more superior.

Efficiency: I raised this last time and I do not see it addressed. Speed is still reported only at batch size 1 (Figure 8), and batched / server-side inference is deferred to future work (Bit 7).

A related point on framing: the closing paragraph of the introduction now claims byte-level LLMs deliver "improved computational efficiency that reduces energy and deployment costs" and mitigate "biases introduced by English-centric subword tokenization." Neither is shown here. Efficiency is demonstrated only at batch size 1 (and is not better at matched compression), and nothing multilingual or bias-related is evaluated at all. This sentence should be rephrased in line with what the experiment results.

Data, Methodology, and Results

Thanks for your improvement. I see some of my concerns are addressed, but some issues still remain, as analyzed below.

1. **The fair, training-matched comparison is still not the main comparison.** Last round I asked for the continued-training baseline, OLMo 3 trained on the same data for the same number of updates, to appear in the main table. This version added a new Figure 3 about it. While the paragraph describing Figure 3 is objective, it is not clear to me why not directly adding the results to Table 1. Especially, given the importance of Table 1, many readers may focus only on this table but omit the added new paragraph, possibly incorrectly comparing approaches that should not be directly compared.

Furthermore, the absolute scores of the continued training is also not accessible anymore, which were accessible in the appendix. Moreover, the new Qwen/Bwen results have no matched baseline at all (there is no "Qwen 3 CT"), so the architecture's contribution is not isolated for the new family either.

Some minor issues on Table 1 and Figure 3:
(a) In Table 1, the bold results are not always the best results. What does bold mean?
(b) The order of Figure 3 is confusing. Why Code(pass@16) and Code(pass@1) are not next to each other and why @16 appears first?

Overall, this revision is not satisfactory to me.

2. **The role of the synthetic character data is acknowledged but still not isolated.** The revision compares Bolmo and Bwen trained on synthetic character data (Appendix D) and uses the CT baseline to separate data from architecture. This is a fair comparison of the architectures. The previous suggestion I gave, training Bolmo without the synthetic character data, could further answer how much of the absolute character-understanding score comes from the synthetic data, but this is optional.

3. **Unexplained changes in the table between the two submissions.** Comparing the Bolmo 7B column across versions, the character scores have moved (CUTE 78.6 → 80.9, EXECUTE 71.6 → 75.4, Char average 75.1 → 78.1) and DS 1000 pass@1 went 14.9 → 15.3, yet every other number in the column is identical. If this is the same Bolmo 7B, why would only the character tasks plus one code benchmark change while everything else stays bit-for-bit unchanged? A retrained model or modified data should move all the numbers at least slightly. Please explain what changed (retraining, evaluation protocol, or the synthetic data) and say which numbers go with which model and evaluation, since this directly affects how the character-understanding results should be read.

4. **Generality.** My generality concern is largely resolved. Yet this part can be improved with the missing matched baseline noted in point 1.

Clarity and Context

The introduction is better: the long list of subword shortcomings has moved to the appendix, and the framing now sits more honestly against existing byte-level work. That addresses the issue I raised before.

Minor Issues

- One new issue: the Boundary Predictor paragraph in the Methods (Appendix A.1) has a broken cross-reference, "...in detail in Section ??".

(Remarks on code availability)

Referee #2

(Remarks to the Author)
I co-reviewed this manuscript with one of the reviewers who provided the listed reports.

(Remarks on code availability)

Referee #4

(Remarks to the Author)
Thank you for the detailed response and the revised manuscript.
I appreciate the authors' careful and substantial revisions.
Overall, many of my previous concerns have been addressed, and the manuscript is now clearer and stronger.
I summarize below how I currently view my previous comments.

A. Summary of the key results: --> resolved.
B. Originality and significance: if not novel, please include reference: --> resolved.
C. Data & methodology: validity of approach, quality of data, quality of presentation: --> resolved.
D. Appropriate use of statistics and treatment of uncertainties: --> resolved.
E. Conclusions: robustness, validity, reliability: --> partially resolved (See Section H).
F. Suggested improvements: experiments, data for possible revision:
F1. Local encoder loss notation: --> resolved.
F2. Stage 2 loss notation: --> resolved.
F3. Exactness of the decoder distillation objective: --> resolved.
F4. Role of the comparison function: --> resolved.
F5. Alternative output-layer techniques and the softmax bottleneck: --> resolved as a clarification.

The newly added footnote 8 explains that the experiments use a full-softmax output layer and why alternative factorizations were not used.
This addresses my concern about the presentation of this point.
I do not think any further experiments are needed to address this particular concern.

F6. Decoding behavior and possible mismatch between the global patch representation and realized byte sequence: --> not fully resolved.

The response is helpful.
My understanding is that the patch representation defines a distribution over possible byte continuations, and that the sampled byte sequence is determined from that representation, much as a sampled token is determined from a hidden state in a standard subword-level language model.

However, unless I have overlooked it, I could not find this point stated clearly in the revised manuscript.
The manuscript explains the distinction between non-causal boundary prediction during prefill and output boundary prediction during decoding.
However, it still does not explicitly address the decoding case I asked about: how readers should interpret the relationship between the current global patch representation, the sequential byte predictions produced by the local decoder/LM head, and the emitted boundary when the sampled byte sequence is not the most likely continuation under that patch representation.

I am not asking for new experiments.
I recommend adding a short explanatory paragraph to Section 2.1 or Appendix A.1.
For example, it could describe what happens during decoding.
It would clarify that there is no additional correction mechanism beyond the usual autoregressive sampling process, and that any apparent "mismatch" is analogous to sampling a non-argmax token in a standard subword-level LM.

If I have misunderstood something, please ignore the above comments.

F7. Definition of TSubwordSuffix: --> resolved.
F8. Parameter count accounting: --> resolved.
F9. Top-p sampling and throughput measurements: --> resolved.
G. References: appropriate credit to previous work?: --> resolved.
H. Clarity and context: lucidity of abstract/summary, appropriateness of abstract, introduction and conclusions: --> partially resolved.

A few broad statements in the abstract, introduction, and conclusion still seem stronger than what the experiments directly establish.
In particular, statements such as
"providing improved computational efficiency that reduces energy and deployment costs"
"mitigating biases introduced by English-centric subword tokenization"
and
"byteifying finally makes byte-level LLMs a practical choice close to the level of subword-level LLMs"
may still read as broader than the experiments directly support.
These may be plausible motivations or implications, but the paper does not directly evaluate them.

I suggest qualifying these statements slightly, for example, by framing them as "suggesting potential advantages for computational efficiency and for domains requiring fine-grained textual understanding," rather than presenting all such advantages as directly demonstrated outcomes.

These comments are suggestions rather than requirements.

(Remarks on code availability)

Open Access This Peer Review File is licensed under a Creative Commons Attribution 4.0 International License, which permits use, sharing, adaptation, distribution and reproduction in any medium or format, as long as you give appropriate credit to the original author(s) and the source, provide a link to the Creative Commons license, and indicate if changes were made.
In cases where reviewers are anonymous, credit should be given to 'Anonymous Referee' and the source.
The images or other third party material in this Peer Review File are included in the article's Creative Commons license, unless indicated otherwise in a credit line to the material. If material is not included in the article's Creative Commons license and your intended use is not permitted by statutory regulation or exceeds the permitted use, you will need to obtain permission directly from the copyright holder.
To view a copy of this license, visit https://creativecommons.org/licenses/by/4.0/

Response to Editor

This letter contains the response to the referees of the Nature submission "Byteifying the Next Generation of Language Models". We thank the editor and the referees for their very constructive and helpful feedback. Here, we provide details about how we addressed the referees' concerns. We have also substantially shortened the paper (now at ≈4966 words per our estimate) and moved content to a new Methods section as appropriate to adhere to the article guidelines.

Response to Referee #1

Thank you for your helpful and thorough review.

> This paper is a significant step of byte-level LLM research, as it substantially lowers the cost of training byte-level LLMs and this is the first time that the performance of byte-level LLMs approaches that of subword models.

We greatly appreciate the positive feedback!

> However, when describing the results, the way the results are presented is significantly biased from an academic point of view.

We have adjusted claims in which the wording may have been too strong in the updated version of the paper (see below).

> The primary results table (Table 1) compares Bolmo 7B against the original Olmo 3 7B, which has not undergone continued training on the Bolmo data mix. The fairer baseline, Olmo 3 CT (continued training on the same data for the same number of parameter updates), is relegated to Appendix A (Table 4).

We have moved the Continued Training (CT) baseline from the Appendix to a dedicated figure in the main paper (Figure 3 in the revised manuscript), which isolates the gap due to the architecture change for every task category. This figure illustrates the large gains on character-level understanding and code pass@16, as well as the slight degradation on general knowledge and the notable degradation on code pass@1.

> The paper's headline result, character understanding, is similarly confounded: the large CUTE improvement (78.6 for Bolmo vs. 56.9 for Olmo 3) is substantially driven by 75M tokens of synthetic CUTE-style training data (Appendix C), and the authors acknowledge that "byte-level models otherwise do not acquire this knowledge through our short training schedule." Olmo 3 CT trained on the same data already reaches 72.9 on CUTE [...] The continued-training baseline should appear in the main table, and a controlled ablation removing the synthetic character data from the Bolmo training mix would be needed to isolate the true architectural contribution.

The aforementioned Figure 3 comparing Bolmo to Olmo 3 CT addresses this problem. It isolates the true architectural contribution since the only difference between the two training procedures — being conducted on the same data for the same amount of parameter update steps — is the architectural change due to byteification. This also clarifies that, in fact, the largest gains are not on character understanding (although character understanding does improve substantially), but code pass@16.

> Similarly, when summarizing the overall performance in the abstract and in the introduction, the paper selects data where Bolmo performs better, and ignore the cases where Bolmo performs worse. For example, as mentioned before, Bolmo performs significantly worse than Olmo 3 on the important metrics pass@1 for most code tasks, yet the introduction only states the improvement on average but ignores the degradation on pass@1.

We have made sure to more carefully comment on results throughout the paper. In particular, we have disaggregated the code task category into code pass@1 and code pass@16 categories, which now shows clearly that there is an improvement in pass@16 at degradation of pass@1. Both are important metrics; higher pass@16, in particular, indicates higher output diversity.

> Furthermore, it is also desirable to further evaluate the generality of Byteification. Byteification is demonstrated exclusively on OLMo 2 and OLMo 3. It remains unclear whether the approach transfers to architecturally different model families (e.g., Llama, Mistral, Qwen), which may differ in embedding structure, attention patterns, or training dynamics.

To address this concern, we have byteified Qwen3 8B Base [YLY+25] into 'Bwen 8B' using the exact same setup (data, hyperparameters) as Bolmo 7B. Bwen exhibits the same trends relative to the source model as Bolmo: large improvements on character understanding and code pass@16, notable degradation in maths and code pass@1, and slight degradation in general knowledge. Bwen 8B also substantially outperforms Bolmo 7B and prior byte-level models due to the higher-performing backbone. This validates our byteification recipe on a model with different architecture (pre-norm vs. post-norm), training procedure (trained on 30T+ tokens vs. Olmo's 6T) and tokenizer.

> The introduction focuses on the shortcoming of the subword models, and lists four main problems of the subword models. [...] [I]n my opinion, the introduction should focus on the shortcomings of existing byte-level models.

We believe it is in principle important to provide context for the reader on the deficiencies of subword-level models in contrast with the conceptual advantages of byte-level models. However, we agree that the balance may have been off toward the subword-level side, so we have adjusted the introduction, as well as moved the detailed exhibition of problems with subword models to the Appendix.

We also thank you for raising minor issues that we have fixed in the revised version, including, as suggested, adding a reference to [ZQKO24] who show that losing character information may be a problem in understanding abbrevation in variable names.

Response to Referee #3

Thank you for your constructive feedback!

> It might be more thorough to continue pretrain the baseline subword-based models on the data mixture, so that you could show the gains of the byte-level model comes from the change in model architecture instead of additional training.

We address this with a baseline consisting of Olmo 3 7B with continued training on the exact same data mix (incl. synthetic data) for the same amount of total parameter update steps. A new figure (Figure 3 in the revised manuscript) compares this model with Bolmo 7B; the only difference between the two is the change in architecture due to byteifying, so the resulting gap is purely due to architecture. Large gains on character understanding and code pass@16 performance persist in this setting.

> The performance gains of the byte-level models are still a bit limited in terms of tasks and domains. Would it be possible to show some improvements in more practical use cases? For example, on multilingual or multimodal tasks.

Our evaluation set is already quite practical: with general knowledge, character understanding, math and coding we cover many skills that make LLMs useful. However, we do agree that multilingual and multimodal applications are relevant; we see these directions as a natural next frontier for byte-level LLMs, and our results on the multilingual EXECUTE benchmark (reported in Table 1) suggest that byte-level LLMs could indeed lead to substantial improvements here. There are some promising avenues to increase equity across languages specific to byte-level LLMs, such as switching the underlying encoding from UTF-8 bytes to e.g. MYTE [LBG+24] or SCRIPT [LA26]. Although we see these directions as out of scope for our initial introduction of byteification, we have added this point to the Future Directions section in the revised manuscript. Additionally, to increase practicability of our artifacts, we have byteified the Qwen 3 8B Base language model [YLY+25]. This model performs substantially better than Bolmo 7B due to the stronger base model, and validates effectiveness of our recipe on another model family.

Response to Referee #4

Thank you for your thoughtful and thorough review.

> I can say that the code preparation is excellent. This code repository would be very valuable for reproducing the experiments in future studies.

We are particularly thankful for your recognition of the code preparation! This is an aspect that we think is especially important but that can go unnoticed.

> [T]he broader conclusion that byte-level LLMs can compete effectively with subword-level LLMs appears somewhat stronger than the comparisons with Olmo 3 directly warrant [...] some of the claims are stronger than the experimental results directly support.

We believe the broader conclusion that byteified LLMs can compete with subword-level LLMs does generally hold; however, we agree that there is nuance regarding the areas of improvement, and it would be incorrect to say that byteified LLMs perform on par with the source LLM in every area (which may have been too easy to miss in the original manuscript). We have thus adjusted our claims and made sure to more carefully discuss the performance differences in the updated version of the paper (see also the response to Referee #1).

> [M]y understanding is that the novelty of the methodological perspective should not be framed as each individual component was newly proposed here in isolation. Rather, the paper's contribution seems to lie in combining and adapting these ingredients into a system specifically suited to byteification.

We agree that a substantial amount of novelty of our approach lies in the particular combination of existing ingredients, but note that some ingredients (e.g., the non-causal boundary prediction) are also novel in and of themselves. To clarify this distinction, we have emphasized novel components in the sections on Architecture and Training vs. those that appeared in some capacity in prior work.

> [I]t remains unclear how well the proposed method generalizes to other model families. The paper would be strengthened if the authors could show that a similar tendency also holds for model families beyond OLMo.

To address this concern, we have byteified Qwen3 8B Base [YLY$^+$25] into 'Bwen 8B' using the exact same setup (data, hyperparameters) as Bolmo 7B. Bwen exhibits the same trends relative to the source model as Bolmo: large improvements on character understanding and code pass@16, notable degradation in maths and code pass@1, and slight degradation in general knowledge. Bwen 8B also substantially outperforms Bolmo 7B and prior byte-level models due to the higher-performing backbone. This validates our byteification recipe on a model with different architecture (pre-norm vs. post-norm), training procedure (trained on 30T+ tokens vs. Olmo's 6T) and tokenizer.

> I think the paper should present clear equations for the statistical significance tests used.

We have added a subsection in the Methods section detailing the precise equations used for statistical tests.

> The definition of the local encoder loss L_E appears to contain a parenthesis error. [...] The notation for the Stage 2 objective is inconsistent (F1, F2).

Thank you, we have fixed these issues in the updated manuscript!

> The claim that the decoder distillation objective $L_{D,Distill}$ is "exact" is not fully convincing from the current presentation. The loss compares patch probabilities using the comparison function $f(\cdot \parallel \cdot)$ with temperature-scaled terms, but it is not obvious from the equations alone why this yields the stated if-and-only-if exactness guarantee. Please provide either a short proof, a clearer derivation, or a more qualified statement (F3).

We have added a subsection on exactness of the Stage 1 distillation objective in the Methods section to clarify this point.

> The role of the comparison function $f(\hat{y} \parallel y)$ is not sufficiently clear (F4).

We have disambiguated the argument order and expanded the reference to make the role of f sufficiently clear.

> [T]he discussion does not consider whether alternative output-layer techniques for reducing softmax cost might affect the frontier comparison. In particular, a range of methods, such as hierarchical softmax, has long been available (F5).

We have restricted these experiments to a full-softmax output layer for two reasons, which we note in a footnote in the revised manuscript: (i) these techniques would require new embedding-initialization methods, since there is not anymore one embedding corresponding to every item in the vocabulary; existing methods like [DdM23] are nontrivial

to adapt to this setting, and (ii) [LGM+23] have noted training instability and slower convergence of hierarchical methods, which is potentially the reason they are not used in any contemporary LLMs (to the best of our knowledge).

> I am not sure I fully understand how decoding behaves when the byte-level output of the local decoder/LM head diverges from the continuation implicitly suggested by the current global patch representation, $\hat{h}$ (F6).

Decoding can, as such, not diverge from the patch representation. To illustrate this point, it may be helpful to consider the subword-level language model case: in this case, the semantics of the last hidden state are given by the probability distribution over tokens it could decode to. Analogously, in a byteified language model, the semantics of the last patch representation ($\hat{h}$) are given by the probability distribution over sequences of bytes it could decode to. As such, mismatch between $\hat{h}$ and the decoded bytes (i.e. how the model 'thinks' it should continue vs. how it ends up continuing) exists only to the same extent as in subword-level models: the patch representation dictates the likely continuations, we then pick a continuation according to some sampling strategy. Although we might not pick the most likely continuation, disagreement of the patch representation and the chosen continuation in the strict sense is not possible since the latter is a function of the former.

> I found the definition of $T_{\text{SubwordSuffix}}(x_{:i})$ somewhat hard to parse, and I think a short, concrete example would help clarify its intended behavior [...] is my understanding correct that the model does not care whether the current word will ultimately be "flower" and instead uses the embedding of "flow" at the position of "w"? (F7).

We have added an example to the definition of the SubwordSuffix function to make it easier to parse, and your understanding is correct.

> A point that may merit clarification is how Bolmo's parameter count should be understood relative to that of the source OLMo model (F8).

The interpretation that byteification removes some parameters (the subword embedding output matrix parameters) and adds some parameters (the local encoder and local decoder parameters) is correct. We report the parameter counts of all tested models in Table 1, and discuss the change in parameter count at the end of section 2.1; it stays comparable in practice (-0.7% to +4.5%).

> [T]he paper does not clearly explain how top-p sampling is implemented in Bolmo's byte-level decoding [...] and it is not clear whether the throughput measurements in Figure 7 are based on greedy decoding or sampling (F9).

Thank you for raising this subtle but important point. We have added information on the sampling algorithms at appropriate locations in the paper. In particular: (i) Sampling algorithms on byteified models are applied over the combined vocabulary of size 512. (ii) For simplicity, throughput estimates are computed with greedy sampling in all cases. Although byte-level models require more frequent sampling, the individual sampling operations require substantially fewer FLOPs since $512 \ll |\mathcal{V}_{\text{subword}}|$; in our experiments, this results in $<5\%$ throughput overhead through sampling for both the subword-level and the byte-level models, so the conclusions from the throughput measurements are unaffected. (iii) As a best attempt to match behavior, sampling settings are equivalent across both the byteified models and the original subword-level models, and always follow the information stated in Table 3.

> The following papers should also potentially be cited when discussing how the motivation, concepts, or methodologies in this paper differ from those of earlier studies.

As recommended, we have added references to [XZZ+25] [DMBB25], and [HI25].

References

[DdM23] Konstantin Dobler and Gerard de Melo. FOCUS: Effective embedding initialization for monolingual specialization of multilingual models. In Houda Bouamor, Juan Pino, and Kalika Bali, editors, *Proceedings of the 2023 Conference on Empirical Methods in Natural Language Processing*, pages 13440–13454, Singapore, December 2023. Association for Computational Linguistics.

[DMBB25] Rares Dolga, Lucas Maystre, Tudor Berariu, and David Barber. From characters to tokens: Dynamic grouping with hierarchical BPE. In Christos Christodoulopoulos, Tanmoy Chakraborty, Carolyn Rose, and Violet Peng, editors, *Findings of the Association for Computational Linguistics: EMNLP 2025*, pages 11154–11162, Suzhou, China, November 2025. Association for Computational Linguistics.

[HI25] Tatsuya Hiraoka and Kentaro Inui. Spelling-out is not straightforward: LLMs' capability of tokenization from token to characters. In Christos Christodoulopoulos, Tanmoy Chakraborty, Carolyn Rose, and Violet Peng, editors, *Findings of the Association for Computational Linguistics: EMNLP 2025*, pages 13340–13353, Suzhou, China, November 2025. Association for Computational Linguistics.

[LA26] Sander Land and Catherine Arnett. BPE stays on SCRIPT: Structured encoding for robust multilingual pretokenization. In *Tokenization Workshop*, 2026.

[LBG+24] Tomasz Limisiewicz, Terra Blevins, Hila Gonen, Orevaoghene Ahia, and Luke Zettlemoyer. MYTE: Morphology-driven byte encoding for better and fairer multilingual language modeling. In Lun-Wei Ku, Andre Martins, and Vivek Srikumar, editors, *Proceedings of the 62nd Annual Meeting of the Association for Computational Linguistics (Volume 1: Long Papers)*, pages 15059–15076, Bangkok, Thailand, August 2024. Association for Computational Linguistics.

[LGM+23] Davis Liang, Hila Gonen, Yuning Mao, Rui Hou, Naman Goyal, Marjan Ghazvininejad, Luke Zettlemoyer, and Madian Khabsa. XLM-V: Overcoming the vocabulary bottleneck in multilingual masked language models. In Houda Bouamor, Juan Pino, and Kalika Bali, editors, *Proceedings of the 2023 Conference on Empirical Methods in Natural Language Processing*, pages 13142–13152, Singapore, December 2023. Association for Computational Linguistics.

[XZZ+25] Zhu Xu, Zhiqiang Zhao, Zihan Zhang, Yuchi Liu, Quanwei Shen, Fei Liu, Yu Kuang, Jian He, and Conglin Liu. Enhancing character-level understanding in LLMs through token internal structure learning. In Wanxiang Che, Joyce Nabende, Ekaterina Shutova, and Mohammad Taher Pilehvar, editors, *Proceedings of the 63rd Annual Meeting of the Association for Computational Linguistics (Volume 1: Long Papers)*, pages 3839–3853, Vienna, Austria, July 2025. Association for Computational Linguistics.

[YLY+25] An Yang, Anfeng Li, Baosong Yang, Beichen Zhang, Binyuan Hui, Bo Zheng, Bowen Yu, Chang Gao, Chengen Huang, Chenxu Lv, et al. Qwen3 technical report. *arXiv preprint arXiv:2505.09388*, 2025.

[ZQKO24] Leonardo Zilio, Shenbin Qian, Diptesh Kanojia, and Constantin Orasan. Using character-level models for efficient abbreviation and long-form detection. In Nicoletta Calzolari, Min-Yen Kan, Veronique Hoste, Alessandro Lenci, Sakriani Sakti, and Nianwen Xue, editors, *Proceedings of the 2024 Joint International Conference on Computational Linguistics, Language Resources and Evaluation (LREC-COLING 2024)*, pages 3028–3037, Torino, Italia, May 2024. ELRA and ICCL.

Response to Referee #1

> [T]he closing paragraph of the introduction now claims byte-level LLMs deliver ”improved computational efficiency that reduces energy and deployment costs” and mitigate ”biases introduced by English-centric subword tokenization.” Neither is shown here. Efficiency is demonstrated only at batch size 1 (and is not better at matched compression), and nothing multilingual or bias-related is evaluated at all. This sentence should be rephrased in line with what the experiment results.

We have rephrased this passage to '*suggesting potential advantages* for [...]' to bring it in line with the experiment results.

> While the paragraph describing Figure 3 is objective, it is not clear to me why not directly adding the results to Table 1. Especially, given the importance of Table 1, many readers may focus only on this table but omit the added new paragraph, possibly incorrectly comparing approaches that should not be directly compared. Furthermore, the absolute scores of the continued training is also not accessible anymore, which were accessible in the appendix.

Thank you for this feedback. In the updated version, we have removed Figure 3 and added the Olmo 3 CT results to Table 1. Bolding in this table is now consistently done to highlight the best score across models with the same backbone (e.g., Bolmo, Olmo 3, Olmo 3 CT).

> Comparing the Bolmo 7B column across versions, the character scores have moved (CUTE 78.6 → 80.9, EXECUTE 71.6 → 75.4, Char average 75.1 → 78.1) and DS 1000 pass@1 went 14.9 → 15.3, yet every other number in the column is identical. If this is the same Bolmo 7B, why would only the character tasks plus one code benchmark change while everything else stays bit-for-bit unchanged?

We have adjusted the formatting of CUTE for consistency with the other benchmarks in the updated version. Previously, it was formatted in a style designed for chat/instruction-tuned models (i.e. generate a response message, given a prompt). We now format it in a style designed for base models (i.e. generate a completion of the given text). This in some cases slightly increased scores, but overall did not have a large effect. The 14.9 → 15.3 change on DS 1000 pass@1 is due to the earlier version mistakenly using the numbers from an earlier evaluation. The model is exactly the same across both versions. We apologize for omitting this information in the initial rebuttal.

We also thank you for letting us know about the broken cross-reference (which we fixed in the new version), and for confirming your concerns on generality, clarity and context were resolved.

Response to Referee #4

Thank you for confirming that many of your concerns have been addressed. We address the two remaining concerns below.

> The manuscript explains the distinction between non-causal boundary prediction during prefill and output boundary prediction during decoding. However, it still does not explicitly address the decoding case I asked about: how readers should interpret the relationship between the current global patch representation, the sequential byte predictions produced by the local decoder/LM head, and the emitted boundary when the sampled byte sequence is not the most likely continuation under that patch representation.

We have added an explanation on this point to the paragraph on the Language Modeling Head in the Bolmo Architecture section in the updated version.

> A few broad statements in the abstract, introduction, and conclusion still seem stronger than what the experiments directly establish. In particular, statements such as "providing improved computational efficiency that reduces energy and deployment costs" "mitigating biases introduced by English-centric subword tokenization" and "byteifying finally makes byte-level LLMs a practical choice close to the level of subword-level LLMs" may still read as broader than the experiments directly support. These may be plausible motivations or implications, but the paper does not directly evaluate them. I suggest qualifying these statements slightly [...]

We have qualified this passage to '*suggesting potential advantages* for [...]' to bring it in line with the experiment results, as also suggested by Referee #1.
